# Operando X‐Ray Diffraction Study of MXene Electrode Structure in Supercapacitors with Alkali Metal Electrolytes

**DOI:** 10.1002/smsc.202500367

**Published:** 2025-10-14

**Authors:** Gui Li, Nicolas Boulanger, Bartosz Gurzęda, Susu Bi, Christoph Hennig, Alexandr V. Talyzin

**Affiliations:** ^1^ Department of Physics Umeå University Umeå S‐90187 Sweden; ^2^ Rossendorf Beamline (BOBL‐BM20) European Synchrotron Radiation Facility (ESRF) 71 Avenue des Martyrs 38000 Grenoble France; ^3^ Helmholtz‐Zentrum Dresden‐Rossendorf Institute of Resource Ecology Bautzner Landstrasse 400 01328 Dresden Germany

**Keywords:** in situ, MXene, operando, supercapacitors, Ti_3_C_2_T_*x*_, two‐dimensional materials, X‐ray diffraction

## Abstract

Ti‐MXene is a promising electrode material for supercapacitors. The layered structure of MXene expands due to swelling in electrolytes allowing the penetration of ions into the interlayers. A study of effects related to the match between the size of cations in hydrated or dehydrated state and the interlayer distance of MXene is performed here using operando X‐ray diffraction (XRD) in capillary‐size supercapacitors with alkali metal chloride electrolytes. The supercapacitors are studied during charging and discharging over several cycles revealing structural changes at both MXene electrodes. Experiments reveal an expansion of the MXene c‐lattice in LiCl, NaCl, and KCl electrolytes (compared to the expansion in pure water) under an increase of applied voltage from 0 to 1 V and structural oscillations related to a change of polarity. The interlayer spacing of MXene remains close to the water‐swollen state in RbCl, CsCl, and NH_4_Cl electrolytes showing no further expansion as a function of applied voltage. Only rather small variations of interlayer spacing are found in H_2_SO_4_ electrolyte during tens of charge–discharge cycles. Analysis of the match between the sizes of ions and the width of MXene interlayers demonstrates that some cations and anions could be inserted into MXene interlayers only in dehydrated state.

## Graphical abstract

Structure of Ti‐MXene electrodes is studied using operando synchrotron XRD in supercapacitors with alkali metal chlorides and H_2_SO_4_ electrolytes. Significant changes in MXene interlayer distance are found during charge/discharge cycling in LiCl, NaCl, and KCl electrolytes. The width of MXene interlayers in RbCl, CsCl, and NH_4_Cl electrolytes remains similar to water‐swollen state, demonstrating effects related to mismatch with ion size.

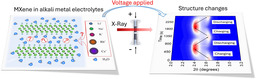

## 1 Introduction

MXenes are materials with a structure consisting of 2D layers of transition metal carbides terminated by a variety of functional groups.^[^ ^1^, ^2^ ^]^ These materials have attracted significant interest in recent years due to many suggested applications, such as energy storage^[^ ^3^, ^4^, ^5^ ^]^ and removal of pollutants from water.^[^ ^6^ ^]^ So far, the most studied MXene is Ti_3_C_2_T_*X*_ produced from Ti_3_AlC_2_ (MAX‐phase) by etching away Al using a variety of methods.^[^ ^3^, ^7^, ^8^, ^9^, ^10^, ^11^ ^]^ The properties of Ti‐MXene are strongly affected by the type of functional groups terminating 2D sheets (such as fluorine, oxygen, or hydroxyls) and depend on the synthesis method.^[^ ^3^, ^9^, ^12^ ^]^

The first methods to produce MXene were to etch aluminum using hydrofluoric acid (HF) or a mixture of HF with LiCl.^[^ ^4^, ^7^, ^13^, ^14^, ^15^ ^]^ This results in a termination of Ti_3_C_2_ layers predominantly by fluorine with a smaller amount of hydrophilic oxygen groups. Excluding water from the synthesis of MXene results in a hydrophobic (e.g., Cl‐terminated) type of MXene synthesized in recent studies of MAX phase etching in molten salts.^[^ ^16^, ^17^ ^]^ The absence of Cl‐terminated MXene swelling in standard aqueous electrolytes is a hindrance to its application in supercapacitor electrodes.^[^ ^18^ ^]^

Etching of the MAX phase using a mixture of LiF and HCl solutions has become increasingly popular in later years.^[^ ^4^, ^19^, ^20^ ^]^ The presence of exchangeable Li‐ions intercalated between the MXene layers and a higher proportion of oxygen groups terminating Ti_3_C_2_ layers makes materials prepared by this method more hydrophilic with “clay‐like” swelling.^[^ ^4^, ^21^ ^]^ The swelling enables penetration of electrolyte ions into interlayer space between 2D sheets of MXene.

The possibility to delaminate MXene into individual 2D sheets, its relatively high surface area, and excellent electrical conductivity of MXene make it very attractive as a material for the preparation of supercapacitor electrodes.^[^ ^22^, ^23^, ^24^, ^25^ ^]^ Electric double layer capacitors (EDLC) store energy by reversible sorption of ions on the surface of the electrode material in the process of charging‐discharging.^[^ ^26^ ^]^ The energy storage in MXene supercapacitors in aqueous electrolytes (e.g., H_2_SO_4_) is typically described as mostly pseudocapacitive, involving fast reversible Faradaic redox reactions at the electrode surface.^[^ ^27^, ^28^ ^]^

High surface area and easy access of electrolyte ions to the surface of 2D MXene flakes are equally essential for both capacitive and pseudocapacitive energy storage mechanisms. The N_2_ BET surface area is typically rather low for powder MXene (e.g., 23 m^2^ g^−1^ in ref. [3]) and not exceeding ≈100 m^2^ g^−1^ even in specially prepared aerogels.^[^ ^29^ ^]^ However, BET surface area is measured at solvent‐free conditions and includes only the outer surface of multilayered MXene flakes. Swelling in aqueous solutions makes the inner surface of each 2D sheet of MXene available for the sorption of ions.

MXene is known to swell in polar solvents with expansion of the c‐lattice and insertion of solvent molecules between individual 2D Ti_3_C_2_T_*x*_ sheets.^[^ ^13^, ^30^, ^31^ ^]^ The width of interlayers filled with electrolyte solutions is then one of the most important parameters that control access of electrolyte ions to the inner surface area of MXene and energy storage parameters of MXene supercapacitors. Spontaneous intercalation of cations into the MXene interlayers in electrolyte solutions was studied by a variety of methods including X‐ray diffraction (XRD),^[^ ^3^, ^13^ ^]^ neutron scattering,^[^ ^32^ ^]^ NMR,^[^ ^33^ ^]^ calorimetry, and AFM.^[^ ^34^ ^]^

However, penetration of cations and anions into interlayers of the MXene structure during the process of supercapacitor operation remains poorly understood.^[^ ^35^, ^36^, ^37^, ^38^ ^]^ For example, the inability of anions to intercalate between the MXene sheets (in 14M LiCl and LiBr electrolytes) reported by N Shpigel et al.^[^ ^39^ ^]^ is expected to also inhibit the penetration of cations in supercapacitor electrodes due to charge balance condition. In the absence of ion penetration, the surface area available for accommodation of both cations and anions should be limited to the outer surface of the material grains which is negligibly small in multilayered materials.

Using operando methods is likely to improve understanding of the energy storage mechanism in MXene‐based supercapacitors which remains not completely clear. The nearly rectangular shape of cyclic voltammetry (CV) curves observed for MXene in acidic electrolytes (e.g., H_2_SO_4_) has been explained by changes in the Ti oxidation state and protonation of oxygen surface groups.^[^ ^27^ ^]^ In situ X‐ray absorption spectroscopy (XAS) was used to detect reactions related to the formation of titanium oxides^[^ ^28^ ^]^ which are likely to form at the surface without forming crystalline titanium oxide phases.^[^ ^40^ ^]^ Gravimetric methods have also been used to study electrochemical intercalation of ions.^[^ ^41^, ^42^ ^]^

The changes of MXene structure are very commonly studied by XRD, revealing a broad range of various phenomena: for example, swelling in polar solvents, changes in interlayer distance due to intercalation of various cations, and degradation due to oxidation.^[^ ^3^, ^30^, ^35^, ^43^, ^44^ ^]^ However, very few operando XRD studies of MXene‐based supercapacitors are available at the moment and most of these are for ionic liquid electrolytes.^[^ ^3^, ^45^ ^]^ Intercalation and deintercalation of Li from organic electrolytes have also been studied in battery setup, most often using organic electrolytes with swelling rather different compared to swelling in water.^[^ ^46^, ^47^ ^]^ The nearly water‐free extremely concentrated LiCl solution is likely to be more similar to ionic liquids in terms of electrolyte migration as a function of applied potential.

The crystalline structure of MXene and the possibility to evaluate the width of solvent‐filled interlayers using XRD make these materials extremely attractive for in situ and operando studies of supercapacitors.^[^ ^37^, ^40^ ^]^ Reversible expansion and shrinking of interlayer distance has been found for MXene in ionic and some aqueous liquid electrolytes as a function of applied potential.^[^ ^28^, ^37^, ^41^, ^48^, ^49^, ^50^, ^51^, ^52^, ^53^, ^54^ ^]^ Slight shrinking of interlayer spacing (0.33 Å) was for example observed at the potential change −1 to −0.2 V for MXene structure in KOH electrolyte.^[^ ^3^ ^]^ Opposite trend was found for the changes of MXene c‐lattice in electrochemical experiments performed in ionic liquids.^[^ ^37^, ^48^ ^]^

The reversible expansion or contraction of interlayer distance as function of applied potential is related to the difference between sizes of cations and anions.^[^ ^48^ ^]^ Therefore, relative sizes of anions and cation can be compared using in situ XRD by observing changes of interlayer distance of the MXene structure under conditions of potential cycling. It should be noted that many in situ studies of electrochemical intercalation of MXene structure have been performed in three‐electrode cells with the counter electrode prepared using other materials, for example, over‐capacitive activated carbon.^[^ ^3^, ^34^, ^41^, ^53^ ^]^

To the best of our knowledge, in situ and operando studies which include characterization of MXene structure on both electrodes of parallel plate supercapacitors in process of charging and discharging are not yet available. The analysis of published data is complicated by differences in the swelling of MXenes synthesized by different methods. Older studies have been performed with MXene prepared by HF etching which shows much less pronounced swelling^[^ ^3^, ^41^ ^]^ in aqueous electrolytes while later studies have been mostly performed with LiF + HCl method and show clay‐like swelling.^[^ ^30^, ^35^, ^43^, ^55^ ^]^ The swelling also strongly depends on the details of postsynthesis washing providing an opportunity to tune the width of interlayers in liquid water in a relatively large interval ≈2–4 Å.^[^ ^43^ ^]^ However, reference tests of MXene swelling in water are not always presented in published studies. Moreover, degradation of MXene during the postsynthesis storage and under conditions of supercapacitor operation needs to be taken into account.^[^ ^56^, ^57^ ^]^

To the best of our knowledge, no systematic in situ and operando XRD studies correlating changes in MXene interlayer spacing with its ability to swell in electrolytes with the size of cations are so far available. Therefore, we performed a set of experiments with alkali metal chloride solutions (LiCl, NaCl, KCl, RbCl, and CsCl) as electrolytes to verify how the size of cations is related to changes in MXene interlayer width during the process of supercapacitor operation. Insertion of alkali metal cations onto MXene‐based electrodes was reported in earlier studies performed using quartz‐crystal microbalance and in situ gravimetric characterization.^[^ ^41^, ^58^ ^]^ In situ hydrodynamic spectroscopy applied to MXene electrodes in alkali metal electrolytes revealed some effects related to a change in water content of MXene electrodes due to the insertion of more hydrophilic (e.g., Li+) and less hydrophilic (e.g., Cs+) cations.^[^ ^41^ ^]^ However, change in the electrode mass, hydration state, and deformation effects were not correlated with XRD structural information in these studies.

Using synchrotron radiation XRD with a submicrometer‐sized beam was used in our earlier study to detect structural changes in MXene electrodes inside in‐plane micro supercapacitors (MS) with H_2_SO_4_‐based gel electrolytes.^[^ ^40^ ^]^ However, using a gel electrolyte is an additional factor which might affect the energy storage mechanism. Moreover, the in‐plane geometry appeared to be poorly compatible with transmission mode XRD.

Operando experiments with parallel plate supercapacitors were performed in this study using a specially designed microscopic cell to monitor structural changes during the process of charging and discharging of Ti_3_C_2_Ti_x_ electrodes. The effect of cation size on the structural breathing of MXene during supercapacitor operation was studied using aqueous alkali metal electrolytes and NH_4_Cl. As a reference, experiments were also performed with H_2_SO_4_ electrolyte, the most used with MXene supercapacitors. Our study reveals size‐related effects related to the cation penetration into the narrow interlayers of the MXene structure expanded by swelling in electrolytes. Strong expansion of MXene interlayer distance and expansion/contraction cycles as a function of applied voltage were found in operando experiments with LiCl, NaCl, and KCl supercapacitors. In contrast, the interlayer distance of MXene in RbCl, CsCl, and NH_4_Cl electrolytes remained similar to water‐swollen state showing only rather minor variations. However, similar supercapacitor performance was found in all alkali metal chloride electrolytes showing no exact correlation with structural changes related to intercalation and de‐intercalation of cations in crystal structure of MXene.

## 2 Experimental Section

### 2.1 Chemicals

Lithium fluoride (LiF, ≈300 mesh), MAX phase (Ti_3_AlC_2_, ≥90%, ≤40 μm particle size), lithium chloride (LiCl, ≥99%), rubidium chloride (RbCl, ≥99.5%), cesium chloride (CsCl, ≥99.5%), and sulfuric acid (H_2_SO_4_, 95–97%) were purchased from Merck (Germany). Hydrochloric acid (HCl) and sodium chloride (NaCl, 99.8%) are from VWR chemicals. Potassium chloride (KCl, ≥99.5%) and ammonium chloride (NH_4_Cl, ≥99.5%) are from Scharlab S.L. (Spain).

### 2.2 MXene Preparation

A PTFE bottle was placed into a water bath at 55 °C. Then, 3 g of lithium fluoride and 30 mL of 12M hydrochloric acid were added to the PTFE container and mixed by magnetic stirrer for 30 min. 3 g of MAX phase was added to the solution slowly. The mixture of powder and solution was stirred for 48 h for complete etching. After the reaction, the solution was washed by centrifugation 6 times (20 000 rpm for 20 min) using deionized water. The solid material was immersed in 1M lithium chloride solution for 25 h. Subsequent washing process was done by centrifugation (15 000 rpm, 10 min) using 6M HCl (≈600 mL) and 2 L DI water. The solid precipitate was collected and freeze dried. All experiments were performed using a freshly prepared batch of MXene within 1–3 weeks after synthesis to minimize possible effects of aging. The powder MXene material was stored under argon prior to experiments at ESRF.

### 2.3 Characterization

In‐house XRD characterization of materials was performed using a Panalytical X'pert X‐ray diffractometer with Cu Kα radiation in Bragg‐Brentano geometry with Soller 0.04 rad slits for incident and diffracted beam, ¼^o^ FDS slit, 2x antiscatter slit, beam width mask 5 mm, and 0.3 to 2 mm receiving slit. Cu Kα average (*λ* = 1.5418 Å) was used for the calculation of d‐spacings of the low‐angle reflections. XPS spectra were recorded using a Kratos Axis Ultra electron spectrometer equipped with a delay line detector analyzer. XPS survey spectra were acquired with a pass energy of 160 eV and high‐resolution spectra at 20 eV. A monochromatic 150 W Al Kα source was used as the excitation source. A hybrid lens system with a magnetic lens, providing an analysis area of 0.3 × 0.7 mm, and a charge neutralizer were used for the measurements. The binding energy scale was adjusted with respect to the C1s line of aliphatic carbon, set at 285.0 eV. Survey spectra were recorded to evaluate element composition of samples and high‐resolution spectra recorded for main elements. All spectra were processed with the Kratos software.

Raman spectra were recorded using a Renishaw Invia Raman spectrometer equipped with 532 nm laser. Zeiss Merlin FEG‐SEM microscope was used for SEM imaging of dry MXene.

In situ and operando XRD measurements were performed at the Rossendorf beamline BM20 (ESRF), using an X‐ray wavelength of *λ* = 0.72769 Å (17 038 eV). The beam size was 100 μm in diameter. Experimental data were collected in transmission geometry using a DECTRIS PILATUS3 × 2 m Si area detector and extracted with the Bubble software. The detector geometry parameters were calibrated with PyFAI using the NIST LaB_6_ standard reference material. Batch peak fitting was used to evaluate evolution of main MXene and MAX‐phase reflections, providing d‐spacing values in most cases with at least 0.01 Å precision. Selected XRD patterns were also analyzed using manual fitting (using PeakFit software).

**Figure 1:**
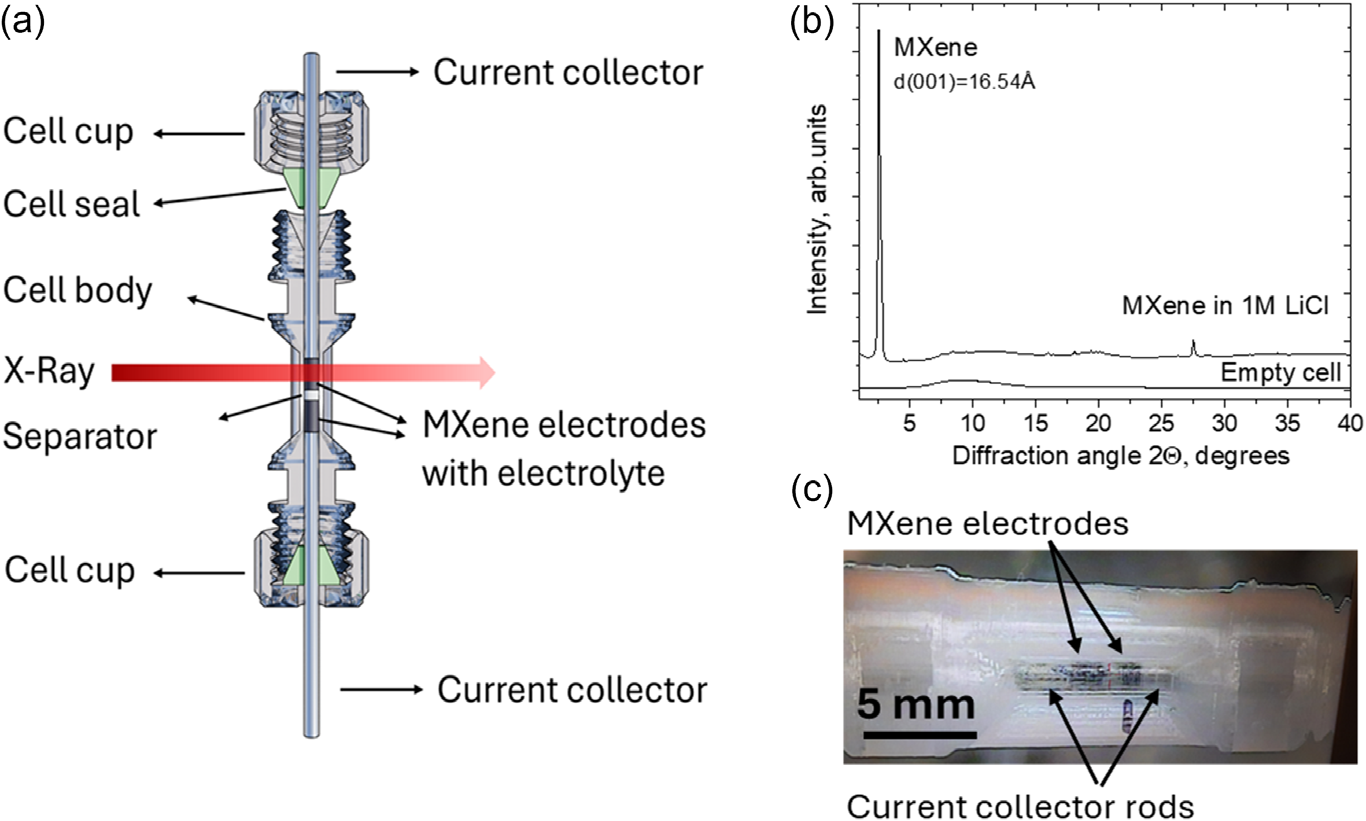
a) Scheme of capillary‐size supercapacitor device designed for operando XRD experiments showing also direction of incident X‐ray beam passing through MXene electrodes toward area detector. b) Example of XRD pattern recorded inside the supercapacitor cell in 1M LiCl electrolyte. c) Image taken from the supercapacitor device during experiments.

The supercapacitor cells were assembled inside a specially designed plastic cell (**Figure** **1**a) which includes a 2 mm (inside) diameter capillary. The cell was prepared by 3D printing using polyethylene terephthalate with glycol (PETG), and stainless‐steel current collectors were exactly fitted to the inside diameter of the capillaries. Parallel plate electrodes were prepared using MXene powder without binder. The thickness of electrodes was about ≈2 mm hand‐compressed using stainless steel current collector rods. Small piece of glass fiber sheet was used as a separator between electrodes. Figure 1c shows an image taken from one of the supercapacitor devices operated with simultaneous recording of XRD data. The incident X‐ray was perpendicular to the capillary walls passing through the MXene electrode parallel to planar surfaces toward 2D area detector.

The exact thickness and weight of electrodes were not precisely controlled due to the microscopic size of the cells. Typical loading was about 1–2 mg. Experiments were performed with 1 M solutions of alkali metal chlorides LiCl, NaCl, KCl, RbCl, and CsCl. Additional experiments were performed with 1M H_2_SO_4_ and NH_4_Cl electrolytes. The plastic capillary is not completely amorphous showing several broad features in diffraction. However, the low‐angle part of diffraction patterns which includes the most important (001) reflection of MXene is available for detailed analysis and free from diffraction features of PETG (Figure 1b). For in situ and operando experiments, the cell was mounted on a standard goniometer head and connected to a Biologic SP‐300 potentiostat (1 μA to 10 A) which was operated remotely from outside the experimental hutch.

Most of the experiments were performed first with XRD recorded on the working electrode in a fully charged state upon increasing the applied voltage from 0 to 1 V with 0.1 V steps followed by a stepwise decrease back to 0 V. Note that in a two‐electrode system, the counter electrode will be negatively charged when a positive voltage is applied to the working electrode. The current was monitored to detect the time required for achieving the full charging/discharging state at each voltage increase/decrease step. A second type of experiments was performed with continuous recording of XRD patterns in operando during two full cycles of slow charging and discharging. Finally, some experiments were performed for prolonged periods of time to record data during tens of charge/discharge cycles. Typically, the data were recorded from the same spot at the working electrode or at the counter electrode.

## 3 Results and Discussion

**Figure 2:**
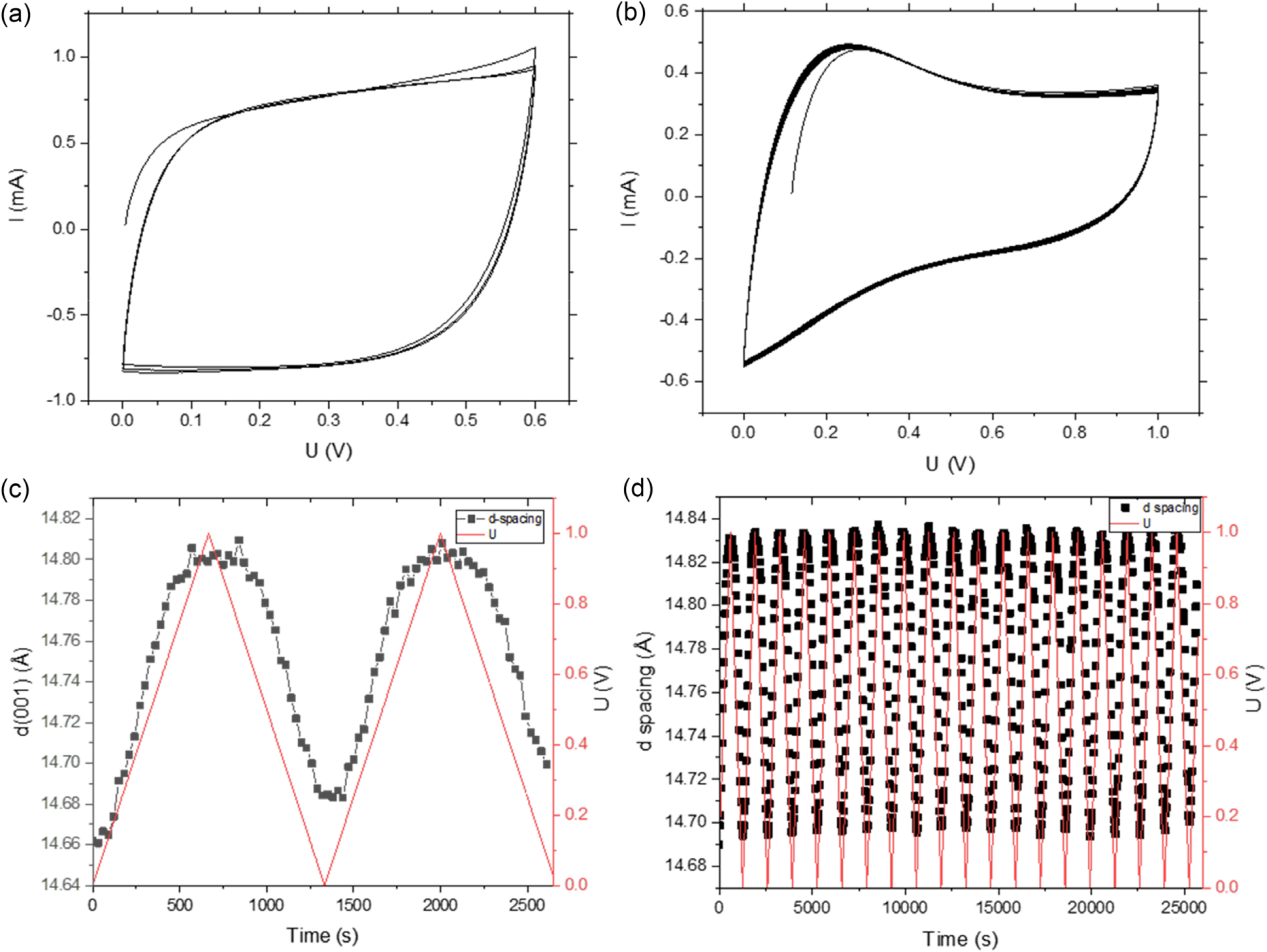
Data recorded using MXene supercapacitors with 1M H_2_SO_4_ electrolyte. a) CV curves recorded immediately after assembling the device. b) CV curves recorded during cycling of the device during operando experiments with XRD. c) Evolution of *d*(001) versus voltage applied to working electrode. d) Evolution of *d*(001) recorded over prolonged period of time from the working electrode.

**Figure 3:**
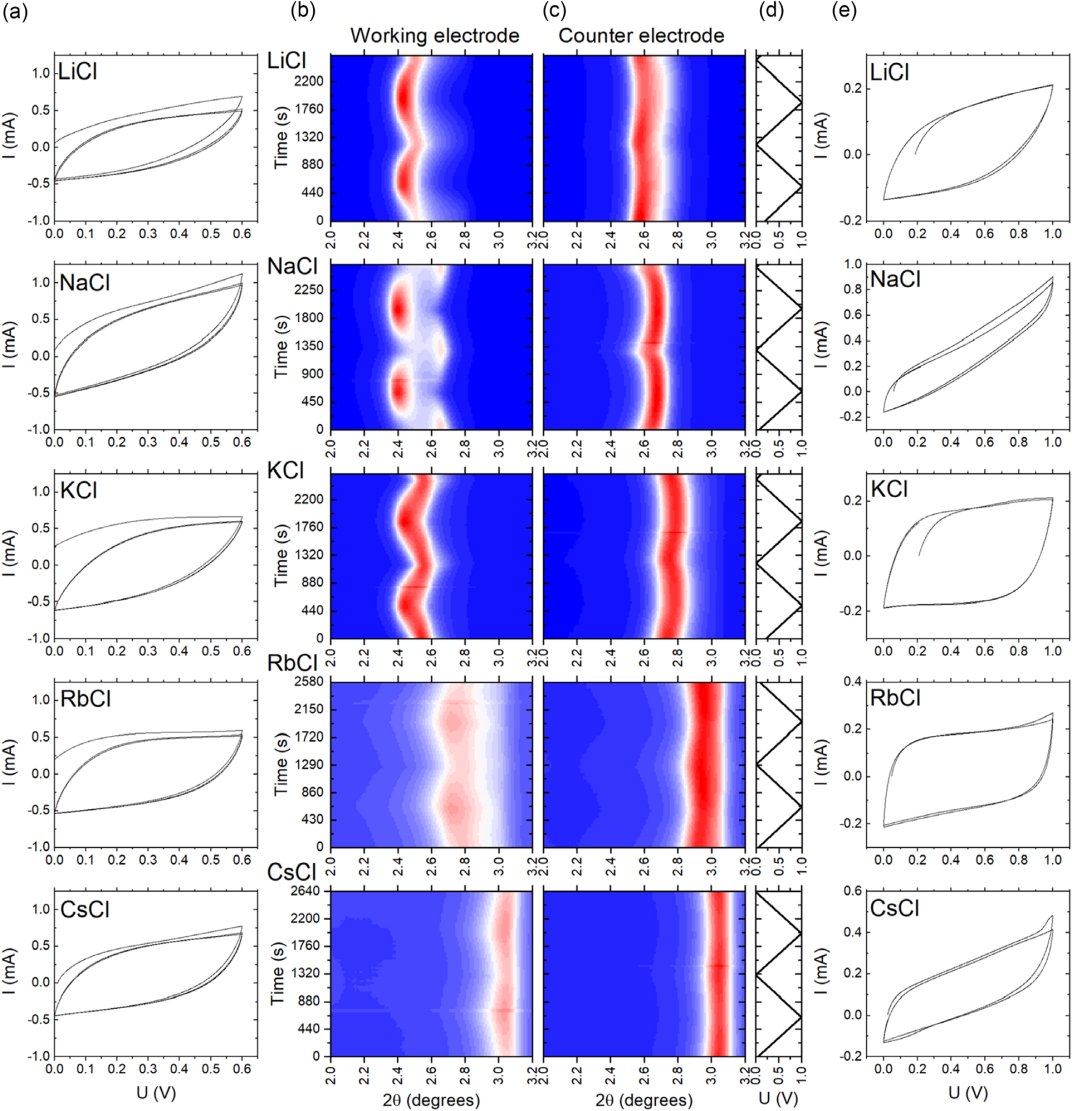
“Heat maps” summarizing operando XRD data recorded in the angle region of the (001) reflection of MXene in 1M LiCl, NaCl. KCl, RbCl, and CsCl electrolytes (*λ* = 0.72769 Å). The data were recorded continuously during charging and discharging experiments in two cycles between 0 and 1 V. Evolution of (001) reflection at working electrode (column b) and at counter electrode (column c) in LiCl to CsCl (top to bottom) set of alkali metal electrolytes is shown in the heat maps. Column d shows time‐dependent change of voltage applied to working electrode over two full cycles. The figure also shows CV loops (column a) recorded prior to XRD testing in 0 to 0.6 V window in order to verify supercapacitor performance and CV recorded during operando XRD tests (0 to 1 V) (column e).

In situ and operando XRD experiments were performed in this study using a specially designed microscopic capillary‐size supercapacitor cell with parallel plate MXene electrodes (Figure 1a,c) in transmission geometry. Despite the rather small size, the devices provided good quality electrochemical data (see examples of CV loops in **Figure** **2** and **3**). Using this cell, we recorded data for MXene supercapacitors with H_2_SO_4_ electrolyte, alkali metal chloride electrolytes, and NH_4_Cl electrolyte (1M in all experiments).

Two types of experiments were performed. The first set of XRD images was recorded at static conditions after a stepwise increase of voltage by 0.1 V from 0 to 1 V (or from 0 to −1 V) and full charging or discharging, controlled by recording current until it completely faded. The data were recorded from the working electrode and from the counter electrode at different applied voltages. The second type of experiments was performed using continuous XRD data recording during slow full charge‐discharge cycles (operando experiments).

The MXene was synthesized using a standard method involving Ti_3_AlC_2_ MAX phase etching with LiF + HCl solution^[^ ^4^, ^40^ ^]^ with all experiments performed within 2 weeks after the material synthesis in order to avoid effects related to possible degradation. Detailed characterization of MXene provided in the SI file (Figure S1–S4, Supporting Information) confirmed the expected structure of the electrode material. The increase in MXene interlayer width in pure water was found to be around 2.3 Å, calculated as the difference between values of *d*(001) in the solvent‐free and water‐immersed state (11.9 and 14.2 Å, respectively) (Figure S1, Supporting Information). Here we use (001) indexing for the main MXene reflection due to the absence of 2D layers ordering in solution intercalated solvate phases. This indexing provides d‐spacing directly corresponding to the inter‐layer distance of the MXene structure.

The swelling in pure water observed for the MXene batch studied here (*d*(001) = 14.2 Å) is smaller than ≈16.5 Å value often reported in the literature for Li‐intercalated material^[^ ^13^, ^30^, ^43^ ^]^ due to postsynthesis washing involving HCl. The washing with HCl was reported to result in smaller saturated swelling of MXene in liquid water (≈14 Å) as compared to LiCl‐washed material (≈16.5 Å)^[^ ^43^ ^]^ due to a smaller amount of Li intercalated into the structure. The smaller expansion of HCl‐washed MXene provides better match with the size of alkali metal cations in dehydrated state.

Nonhydrated alkali metal cations increase in diameter in absence of hydration starting from the smallest Li^+^ (1.36 Å) and reaching 3.34 Å for Cs^+^. The size of H_3_O^+^, which is likely to be the main charge carrier in H_2_SO_4_ electrolyte, is about 2.0 Å. The size of hydrated Li, Na, and K cations is larger than the size of hydrated Rb and Cs cations, thus showing an opposite trend compared to sizes of nonhydrated cations.^[^ ^59^, ^60^ ^]^ This trend reflects more hydrophilic nature of Li, Na, K cations and “hydrophobic” nature of smaller Rb^+^ and Cs^+^ cations which is reported to affect, for example, hydration of MXene from the vapor phase.^[^ ^61^ ^]^

Therefore, the set of alkali metal electrolytes studied here allows to verify how cations with the size smaller (Li, Na, K) and larger (Rb, Cs) than the width of MXene interlayers expanded by swelling in pure water (2.3 Å) are inserted into the MXene structure as a function of applied voltage.

### 3.1 MXene Structure in Supercapacitors with H_2_SO_4_ Electrolyte

The data collected during operando experiments with the MXene supercapacitor in H_2_SO_4_ electrolyte are summarized in Figure 2. This electrolyte is the most common in experiments with Ti_3_C_2_T_*x*_ supercapacitors, providing stable performance and good energy storage parameters. The MXene supercapacitor assembled inside the capillary cell showed expected electrochemical performance (Figure 1a,b) with the shape of CV curves close to square if recorded in a 0 to 0.6 V voltage window and deviating from a square shape if the voltage window is increased to 1 V.

Our experiments demonstrated only rather small variations in the interlayer distance of the MXene structure upon operation of supercapacitors with H_2_SO_4_ electrolyte. The interlayer distance of MXene (*d*(001) spacing) at the working electrode was found to expand from ≈14.6 Å up to ≈14.8 Å during an increase of voltage up to 1.0 V and reversibly decrease back during the 1 to 0 V half‐cycle (Figure 2c). Similar changes were observed at static conditions upon stepwise increase of applied voltage from 0 to 1 V with 0.1 V steps (Figure S7a–d, Supporting Information). The changes of the *d*‐value observed at the counter electrode appeared to be even smaller (within 0.03 Å, Figure S7b, Supporting Information).

An additional experiment was performed over a prolonged period of time and tens of charge‐discharge cycles. The interlayer spacing of the working MXene electrode recorded over this prolonged time remained to be stable and reversible even after tens of cycles (≈14.8 Å at 1 V and ≈14.7 V at 0 V, Figure 2d).

The *d*(001)‐spacing changes were also recorded during stepwise voltage changes from 0 to −1 V. The *d*(001) value decreased by ≈0.3 Å at −1 V and remained nearly the same (irreversibly) when the voltage was decreased back to 0 V (Figure S7e–h, Supporting Information).

Our results demonstrate that the interlayer distance of MXene in the working electrode expands when a positive voltage is applied and remains unchanged at the counter electrode. When a negative voltage is applied to the working electrode, the interlayer distance decreases by ≈0.3 Å while counter electrode structure expands by ≈0.1 Å. The overall change in *d*(001) observed between the data recorded at the 1 and −1 V is about 0.7 Å at working electrode and ≈0.1 Å on the counter electrode. The difference between *d*(001) values at working and counter electrodes was also verified in a linear scan along the capillary performed after a long cycling experiment. Using a 0.2 mm scan step, the value of about 14.8 Å was recorded at the working electrode and ≈14.3 Å on the counter electrode.

It can be summarized that the MXene supercapacitor showed reversible and stable performance in H_2_SO_4_ electrolyte for a 0 to 1 V voltage window during at least 20 cycles. The XRD patterns recorded at the starting point of cycling and after 20 cycles (≈7.5 h) are nearly identical. Analysis of these patterns does not reveal the formation of *crystalline* TiO_2_ as a result of cycling (Figure S6, Supporting Information). The interlayer distance of MXene structure remained similar to the value found in pure water (14.2 Å) slightly increasing and decreasing during the voltage changes. No signs of MXene degradation or degradation of energy storage performance of supercapacitor were found despite using applied voltage up to 1 V (Figure 2).

The change in *d*(001) of about 0.12 Å and successful operation of supercapacitor devices in H_2_SO_4_ indicate that the width of interlayers was sufficient for penetration of both cations and anions. However, the diameter of the sulphate anion is about 4.8 Å in dehydrated state and ≈8.6 Å in the hydrated state, which are larger than the width of MXene interlayers calculated as the difference between water saturated and water free state (≈2.5 Å). Alternative method to calculate width of MXene interlayers is to consider difference between Li‐free state (≈10.5 Å) and water swollen state (14.2 Å) providing ≈3.7 Å value.^[^ ^21^ ^]^ Considering the size relations between the width of MXene interlayers and size of ions, only cations (H_3_O^+^) are sufficiently small to enter and escape the space between 2D sheets filled with water even in the interlayer width is ≈3.7 Å.

Successful operation of MXene supercapacitors with H_2_SO_4_ electrolyte suggests that both anions and cations need to be accommodated on the opposite electrodes in order to fulfil the charge balance condition. The successful operation of supercapacitors for the specific MXene batch used in our experiments was also confirmed using a reference test with a standard Swagelok type cell (Figure S21, Supporting Information) in 1M H_2_SO_4_ electrolyte with specific capacitance exceeding 100 F g^−1^. This value is in good agreement with literature data.^[^ ^62^ ^]^

The puzzling effect of size mismatch between sulphate anions and the width of MXene interlayers could possibly be related to random interstratification of differently intercalated MXene interlayers.^[^ ^31^ ^]^ This interpretation suggests that *d*(001) value does not accurately reflect the true interlayer distance of MXene structure but instead relates to the proportion between intercalated and not intercalated galleries if interlayers stacked randomly. It means that experimentally recorded *d*(001) value can be smaller than the true interlayer distance of ion‐intercalated MXene lattice. The question about match of ion diameters and width of MXene interlayers will be addressed in more detail below in the discussion section.

Results presented above are in agreement with our earlier operando experiments performed with in‐plane micro supercapacitors with an H_2_SO_4_‐based gel electrolyte.^[^ ^40^ ^]^ However, using bulk parallel plate MXene electrodes (rather than thin film electrodes) and a gel‐free aqueous electrolyte allowed us to detect small variations of MXene interlayer distance and to provide a lot more detailed analysis of structural changes.

### 3.2 MXene Structure in Supercapacitors with Alkali Metal Chloride Electrolytes

The next set of experiments was performed with MXene supercapacitors in 1M alkali metal chlorides and NH_4_Cl solutions. The structural changes of MXene electrodes were found to be rather different depending on the size of alkali metal cations (Figure 3). The CV data recorded in 0–0.6 V voltage window showed similar performance for all electrolytes. An exact evaluation of energy storage parameters was not possible due to the absence of exact control over the mass of the micro electrodes. However, the CV loops (Figure 3) demonstrate similar shape and approximately similar area for all electrolytes. The shape of CV curves is also in good agreement with data recorded using standard type of supercapacitor devices (Figure S15–S22, Supporting Information). Therefore, we estimate specific capacitance of MXene electrodes in our experiments on the level of about 45–60 F g^−1^.

It should be noted that swelling of MXene in 1M solutions of alkali metal chloride salts reveals strong differences even without applied voltage. First group of electrolytes (LiCl, NaCl, and KCl) showsstrong expansion of MXene interlayers (in the absence of applied potential) due to spontaneous cation intercalation. MXene immersed in these electrolytes shows *d*(001) values of 16.49 and 16.03 Å (for LiCl and NaCl respectively). That is ≈4–4.5 Å increase compared to the value found in water‐free state and also ≈2–2.5 Å increase compared to the value found in pure water (Figure S5, Supporting Information). Notably, MXene in KCl showed major (001) reflection at 13.97 Å with small shoulder at ≈16.4 Å indicating inhomogeneous hydration. The *d*(001) value of MXene immersed in all other electrolytes (RbCl, CsCl, NH_4_Cl) was found to be similar (13.8–14.0 Å) to swelling in pure water. These results are in agreement with earlier studies of cation‐exchanged MXene swelling in water vapors for Li‐ and Na‐MXenes while for K‐Mxene swelling was absent.^[^ ^14^ ^]^ The swelling stronger than in water can be considered as evidence of spontaneous intercalation of Li^+^, Na^+^, and K^+^ cations into MXene interlayers, while the absence of this effect is evidence for the absence of Rb^+^ and Cs^+^ intercalation.

Distinctly different structural changes were observed in MXene electrodes in these two groups of electrolytes (LiCl, NaCl, KCl, and RbCl, CsCl, NH_4_Cl) also as a function of applied voltage. Strong changes of *d*(001) as a function of applied voltage were observed only in LiCl, NaCl, and KCl electrolytes.

Figure 3 shows “heat maps” summary of XRD data collected for MXene electrodes in operando experiments where the voltage was changed between 0 and 1 V in two complete cycles. The XRD patterns were continuously recorded at the working electrode at the same spot and in separate cycles at the counter electrode. These data are also supported by experiments where XRD patterns were recorded at static charge‐equilibrated conditions at voltages from 0 to 1 V (changed with 0.1 V steps) followed by a decrease back to 0 V, except that the magnitude of structure changes between maximum and minimum applied voltage was somewhat smaller in operando experiments, most likely due to kinetic reasons. **Table** **1** shows *d*(001) values recorded at the end points of cycles at 0 and 1 V for MXene in all electrolytes.

**Table 1:** Interlayer distance of MXene electrodes (*d*(001)) recorded at maximal (1 V) and minimal (0 V) applied voltage during cycling performed in two ways: first one at static conditions after introducing 0.1 V increments and second (value in brackets) recorded directly under operando charge–discharge cycles.

Electrolyte 1M	*d*(001), Å at working electrode			*d*(001), Å at counter electrode			
	1 V	0 V	Δ*d*(001), Å1–0 V		1 V	0 V	Δ*d*(001), Å1–0 V
H_2_SO_4_	14.79 (14.81)	14.65 (14.69)	0.16 (0.12)		14.29 (14.05)	14.31 (14.01)	−0.02 (−0.04)
LiCl	17.0 (17.1)	16.3 (16.6)	0.7 (0.5)		16.21 (15.82)	16.76 (16.01)	−0.65 −0.19
NaCl	17.3 (17.3)	15.6 (15.7)	1.7 (1.4)		15.7 (16.3)	17.0 (17.4)	−1.3 (−1.1)
KCl	16.9 (16.9)	16.4 (16.3)	0.5 (0.6)		15.8 (16.9)	16.2 (16.4)	−0.4 (−0.5)
RbCl	(15.2) (14.1)a)	(14.9) (13.9)a)	(0.3) (0.2)a)		14.21	14.12	+0.09
CsCl	13.96 (13.85)	14.0 (13.76)	0.04 (0.09)		13.88 (13.69)	13.72 (13.72)	−0.04 (−0.03)
NH_4_Cl	14.49 (14.45)	14.24 (14.3)	0.25 (0.15)		14.04	14.43	−0.19

a) Weak additional component observed in RbCl electrolyte during operando cycles.

**Figure 4:**
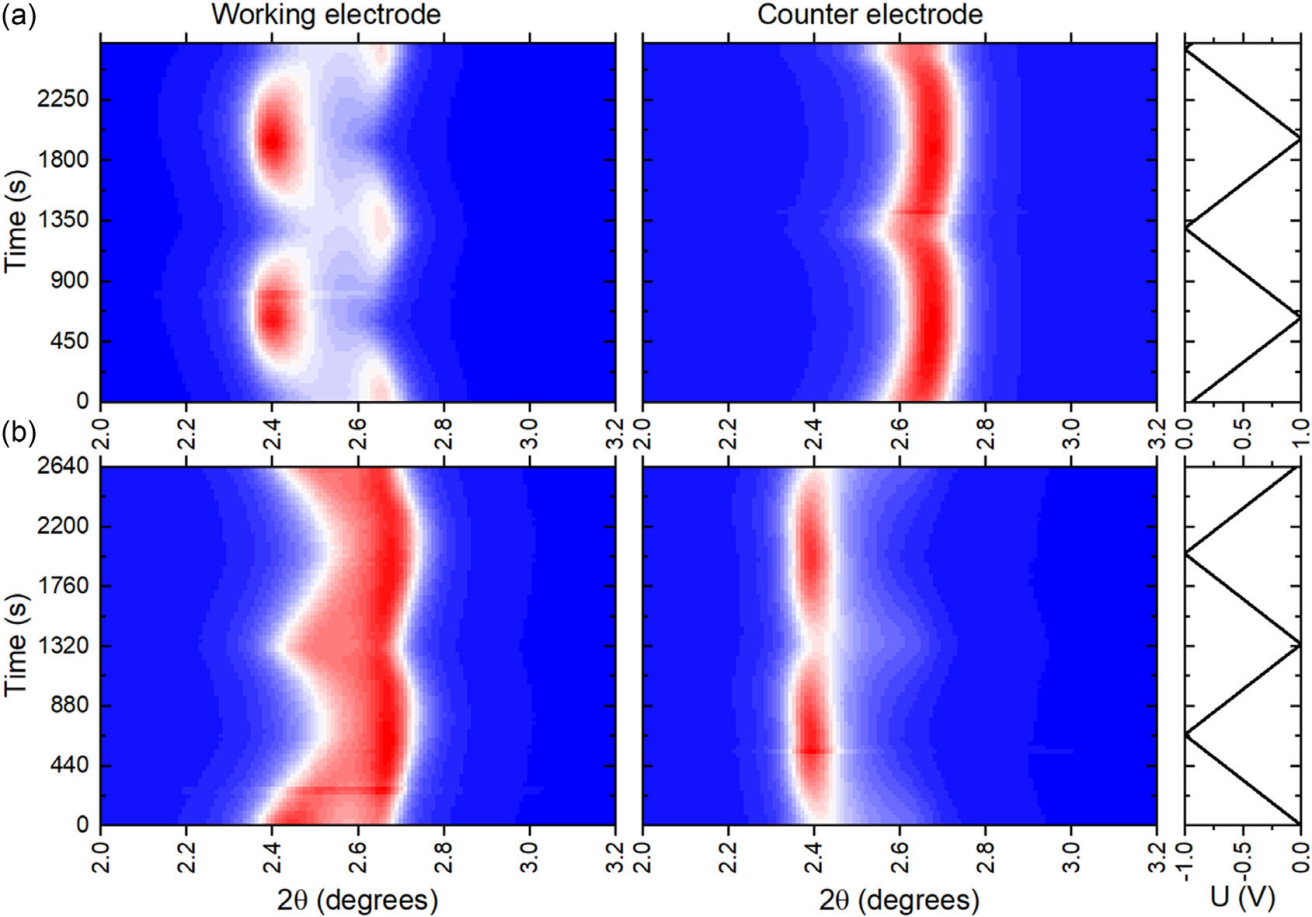
The XRD “heat maps” showing position of (001) reflection of MXene structure in NaCl electrolyte for the working electrode and counter electrode as a function of voltage applied, a) 0 to 1 V and b) 0 to −1 V, to working electrode over two complete charge discharge cycles.

Analysis of XRD data shows a general trend for all electrolytes: expansion of interlayer spacing at the working electrode when positive voltage is applied (from 0 to 1 V) and the shrinking of inter‐layer distance when the voltage goes back to zero or when negative voltage is applied (up to −1 V) (**Figure** 3 and **4**). Opposite trend is found at the counter electrode (applied voltage 0 V) where the largest c‐lattice expansion is found at 0 V (at working electrode) and the smallest *d*(001) value is at 1 V (at working electrode). This trend corresponds to the size of inserted anions larger than the size of withdrawn cations.

However, the magnitude of changes and initial values of *d*(001) at 0 V are very different (Figure 3 and Table 1) and specific for each electrolyte. The structure of MXene is significantly expanded (compared to the 14.2 Å value found in pure water) in experiments with LiCl, NaCl, and KCl electrolytes. The 0 V value of *d*(001) recorded at the working electrode after the first cycle is 16.4 Å for LiCl, and about 16.3 Å for NaCl and KCl. At 1 V, the *d*(001) spacing increases up to 17.03, 17.30, and 17.08 Å for LiCl, NaCl, and KCl, respectively. The changes are much smaller at the counter electrode. The XRD data recorded for MXene in NaCl electrolyte are especially remarkable, showing two rather distinct (001)‐reflections at 0 and 1 V, while for other electrolytes, the changes with applied voltage are more continuous (Figure 3).

In contrast, a large expansion of the MXene c‐lattice is absent in electrolytes with the largest cation size (CsCl) and is rather small in the RbCl electrolyte (Table 1). An additional experiment with NH_4_Cl electrolyte also revealed *d*(001) values close to the one observed in pure water (14.2 Å at 0 to 14.5 Å at 1 V). The width of MXene interlayers remains similar to the value found in pure water for this group of electrolytes (CsCl, RbCl, and NH_4_Cl) indicating the absence of cation intercalation. However, the CV loops recorded during the cycling demonstrate that supercapacitor devices with MXene electrodes still have a reasonably good performance even with RbCl, CsCl, and NH_4_Cl electrolytes which is rather puzzling if the cations are not penetrating the interlayers.

Increasing the voltage window from 0 to 0.6 V (Figure 3a) to 0 to 1 V (Figure 3e) results in a change in CV loop shapes, especially pronounced for NaCl electrolyte. The change is typically attributed to redox reactions on the surface of MXene sheets. Note that no reflections from crystalline TiO_2_ were found in our XRD data even after cycling up to 1 V.

Table 1 provides values of *d*(001) for MXene in all studied electrolytes at the end points of charge discharge experiments with a step‐like increase and decrease of voltage applied to the working electrode. In these experiments, the XRD patterns were recorded at static conditions on every step after allowing the charging /discharging current to fade close to zero.

XRD data were also collected in operando experiments with negative voltage applied to the working electrodes for some electrolytes (Figure 4 for NaCl) and for all electrolytes under static conditions with step‐like changes of voltage from 0 to −1 V and back to 0 V (Figure S7–S12, Supporting Information, and **Table** **2**).

**Table 2:** Interlayer distance of MXene electrodes (*d*(001)) recorded at maximal (−1 V) and minimal (0 V) applied voltage during cycling performed at static conditions after introducing 0.1 V increments.

Electrolyte 1M	*d*(001), Å at working electrode			*d*(001), Å at counter electrode		
	−1 V	0 V	Δ*d* (001) −1 to 0 V	−1 V	0 V	Δ*d*(001) −1 to 0 V
H_2_SO_4_	14.08	14.10	+0.02	14.25	14.23	−0.02
LiCl	15.84	15.67	−0.17	16.81	16.28	−0.53
NaCl	15.50	15.48	−0.02	17.42	16.64	−0.78
KCl	15.78	15.74	−0.04	16.64	16.17	−0.47
RbCla)	14.6	14.5	−0.1	14.4	13.75	−0.65
CsCl	13.78	13.76	−0.02	13.84	13.72	−0.12
NH_4_Cl	14.12	14.13	0.01			

a) Data available only for operando cycles.

The data recorded for MXene in NaCl electrolyte with positive and negative voltage applied to the working electrode during two complete cycles are compared in Figure 4. As expected, the changes in MXene interlayer spacing are opposite for positive and negative voltage windows. However, the change in *d*(001) seems to be more continuous during cycling in 0 to −1 V voltage window. The maximal c‐lattice expansion is found at 1 V when positive voltage is applied and at 0 V when negative voltage is applied to the working electrode. Moreover, the *d*(001) value drops to 15.5 Å at −1 V, thus changing between 1 and −1 V by ≈1.6 Å. The changes between *d*(001) values recorded at +1 and −1 V states in static in situ experiments are tabulated for all electrolytes in Table 1. The changes recorded at static conditions are only slightly larger compared to the operando experiments shown in Figure 3 and 4.

**Figure 5:**
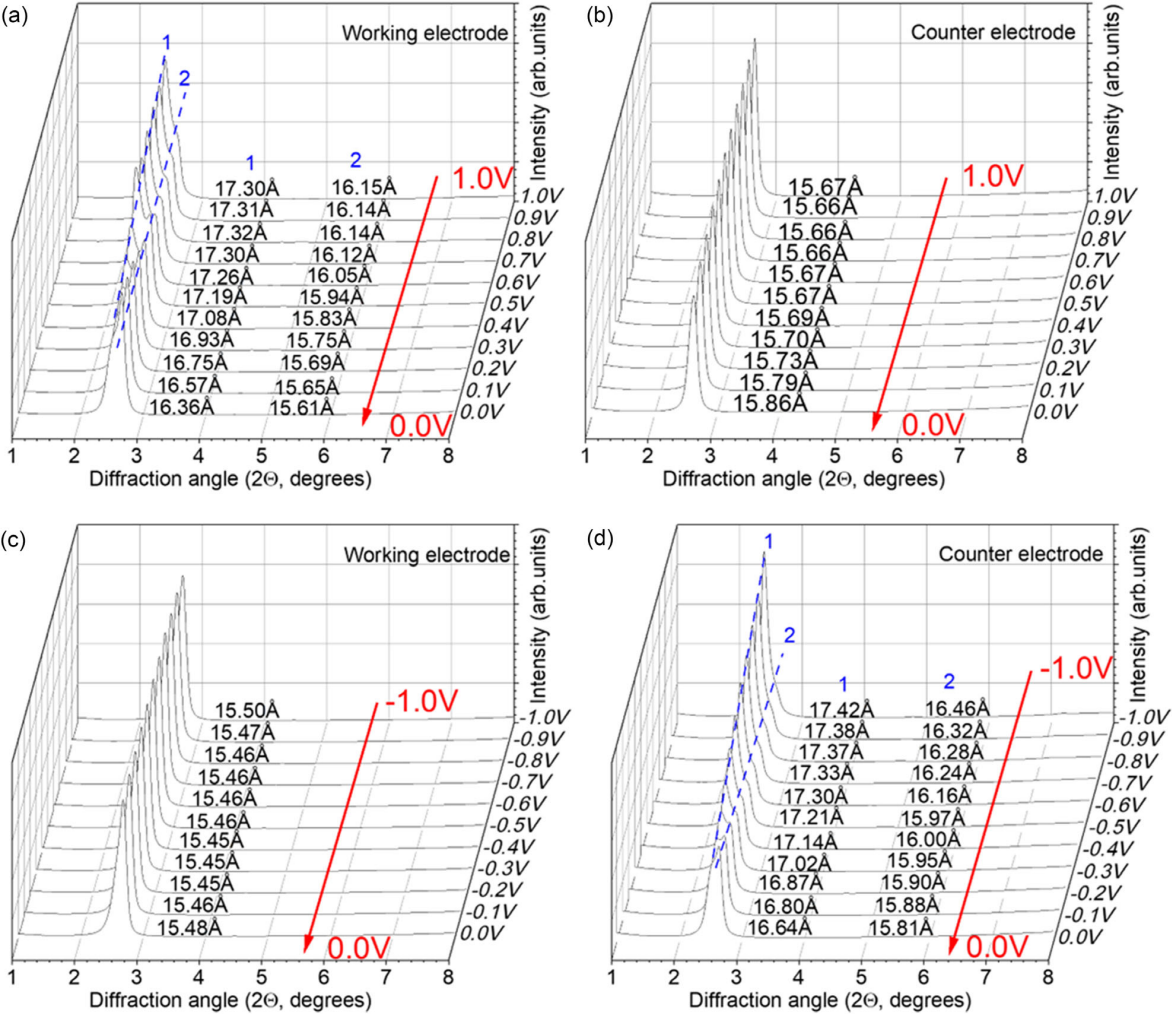
XRD data recorded for MXene in 1M NaCl electrolyte at static conditions (after charge equilibration controlled by recording current) in two experiments with stepwise changes of applied voltage by 0.1 V: from 1 to 0 V at a) working electrode and b) counter electrode; change from −1 to 0 V at c) working electrode and d) counter electrode. Note that arrows show the change of voltage at working electrode also for data recorded at counter‐electrodes.

It is also important to note the strong difference in *d*(001) values recorded at the counter electrodes when +1 and −1 V voltages are applied to the working electrode. The evolution of *d*(001) for MXene in NaCl recorded for 1–0 V voltage window and −1 to 0 V at static conditions with 0.1 V steps is shown in **Figure** **5** (see also Figure S9, Supporting Information, for the complete set of data).

The expansion of the c‐lattice is maximal at 1 V (≈17.3 Å) and minimal at −1 V (15.5 Å) at the working electrode. The *d*(001) splits into two distinct components at the working electrode when positive potentials are applied (Figure 5a) and at the counter ‐electrode when negative potential is applied to the working electrode (Figure 5d). Considering only the major component of (001) reflection, the value observed at 1 V at working electrode is very close to the value observed at −1 V (applied to the working electrode) at the counter electrode (17.30 and 17.42 Å, respectively). There is only one relatively sharp reflection on the counter ‐electrode with a small variation of *d*(001) in the ≈15.5–15.8 Å range when positive voltage is applied to working electrode (Figure 5b) and at working electrode when negative voltage is applied (Figure 5c).

The analysis of XRD data recorded for the NaCl electrolyte leads to the following conclusion: the MXene structure expands when anions are driven into inter‐layers (i.e., positive potential at the working electrode and at the counter ‐electrode at negative potential) and are replacing cations. We therefore suggest that the two (001) reflections observed in Figure 5a,d correspond to 1) MXene interlayers intercalated with Cl^−^ anions, showing a (001) reflection with a maximum spacing of 17.3–17.4 Å, and 2) a part of the electrode that remains nonintercalated, exhibiting a (001) reflection at 15.8–16.5 Å, even at maximal applied voltage.

Intercalation of anions on one electrode must correspondingly be compensated by intercalation of cations on the other electrode. According to the data shown in Figure 5b,c, intercalation of cations occurs without a split in the (001) reflection, which is evidence of homogeneous intercalation throughout the whole electrode at all values of applied voltage. The results of experiments with negative voltage applied to the working electrodes are summarized in Table 2 based on experiments with XRD recorded at static conditions (Figure S7–S12, Supporting Information) with stepwise changes of voltage. The values shown in the table correspond to the second half of the cycle (the value for 0 V is recorded after a change of voltage from −1 V). Only the operando data were recorded for the RbCl electrolyte and used in the table.

Cycling of MXene in RbCl was also tested for a voltage window from +1 to −1 V (Figure S13, Supporting Information) showing a difference in *d*(001) at the working electrode of about 0.4–0.5 Å but also some irreversible changes over several cycles. The maximal and minimal values of *d*(001) were observed to decrease over time changing from 14.27 to 14.73 Å in the first cycle to 14.06–14.51 Å in the sixth cycle. The CV loops recorded over the full interval of applied voltage also clearly demonstrated bumps due to redox reactions (Figure S13, Supporting Information). These features were absent in experiments performed in 0–0.6 V voltage window indicating nonfaradaic energy storage.

Some of our data can be explained only by taking into account nonhomogeneous intercalation of Cl^−^ into the MXene structure. It is evident from the data shown in Figure 2 and especially clearly for the NaCl electrolyte. The structural changes observed at the working electrode during the cycling of MXene in NaCl electrolyte between 1 and 0 V correspond to the coexistence of two states with a difference of about 1.8 Å. The relative intensity of the two *d*(001) reflections changes as a function of voltage but both peaks are present at 0 and 1 V. Therefore, chlorine anions are intercalating only part (major at 1 V) of the whole MXene electrode. It is known that the effects of interstratifications are commonly found in hydrated/solvated MXene.^[^ ^31^ ^]^ Therefore, changes of *d*(001) in a system with random stacking of differently intercalated interlayers are not necessarily in perfect agreement with the size of the intercalated species. The changes of *d*(001) can be smaller depending on the proportion between the relative amounts of differently intercalated interlayers. The changes of *d*(001) as a function of applied voltage are more gradual for MXene in LiCl and KCl electrolytes not showing sharply different structures, thus indicating even stronger effects of random interstratification in the intercalation of interlayers.^[^ ^31^ ^]^

## 4 Discussion

The data shown above provide operando information about the structure of MXene electrodes in supercapacitors with the same anions (Cl^−^) and different cations (alkali metals and ammonium).

Our study is focused on the change in the interlayer width of the MXene structure induced by applied positive and negative voltage inside real parallel plate supercapacitor devices. Intercalation of cations and anions into the MXene structure is expected to affect the width of interlayers. The commonly accepted mechanism of energy storage in EDLC supercapacitors suggests that high energy storage capacity is related to the high surface area of electrode materials. High surface area in MXene electrodes is commonly considered to originate from the access of ions into the interlayer expanded by swelling in electrolyte solutions. Therefore, analysis of changes in interlayer width is expected to not only provide information about the intercalation of ions into the MXene structure but also provide valuable information about energy storage mechanism and possible ways to improve the energy storage capacity of MXene supercapacitors.

**Figure 6:**
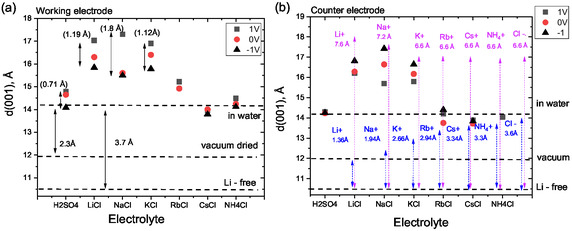
Interlayer distance *d*(001) of MXene electrodes recorded in several electrolytes (1M) under operation of supercapacitor devices at 0, 1, and −1 V. a) Working electrodes and b) counter ‐electrodes. Blue and pink arrows correspond to the diameters of cations and Cl anions in dehydrated and hydrated state, respectively. Horizontal black dashed line indicates *d*(001) values recorded from vacuum‐dried sample of MXene powder and the same sample immersed in water. Also expected *d*(001) value for Li‐free MXene is shown as a reference. The 2.3 Å and 3.7 Å are the values of interlayer width of MXene immersed in water calculated by assuming vacuum‐dried and Ll‐free structures as a reference (see text).

Experimental data collected for supercapacitors in all electrolytes at maximal and minimal applied voltage (+1 and −1 V) are summarized in **Figure** **6**. It shows experimentally observed *d*(001) spacings, width of MXene interlayers estimated using two different methods (2.3 and 3.7 Å), and diameters of ions in both hydrated and nonhydrated state (Figure 6b).

According to the data shown in Figure 6, the interlayer distance of MXene in all studied aqueous electrolytes expands when positive potential is applied and shrinks at more negative potentials. That is in agreement with an early in situ XRD study of MXene intercalation with ions, which was performed using HF‐etched material.^[^ ^3^ ^]^

The difference between the *d*(001) values observed for MXene at +1 and −1 V must be related to replacement of cations with anions. Therefore, the magnitude of this difference provides information about relative diameters of cations and anions intercalated into MXene c‐lattice. The data shown in Figure 6 demonstrate that the size of anions is larger than the size of cations for all studied aqueous electrolytes.

However, the magnitude of changes observed in our experiments with chloride salt solutions is very different and not always clearly correlated with sizes of hydrated or dehydrated cations and anions (Figure 6b). Two sharply different groups of electrolytes can be distinguished using Figure 6: electrolytes providing strong change of swelling compared to pure water (LiCl, NaCl, KCl) and electrolytes with swelling similar (or even slightly smaller) than in pure water (RbCl, CsCl, NH_4_Cl). Intercalation of electrolyte ions is obviously affecting interlayer distance of the MXene structure also when voltage is applied to MXene electrodes in supercapacitors. The first group of electrolytes shows strong changes of *d*(001) as a function of applied voltage, while second group of electrolytes does not reveal any obvious effects of ion intercalation or deintercalation.

The difference between the diameter of the chlorine anion and the diameter of Na^+^ cation in dehydrated state (≈1.7 Å) is in agreement with the observed change of the experimental value of *d*(001) by 1.8 Å. The difference between the diameters of Cl^−^ and K^+^ (0.9 Å) is also in reasonable agreement with experimentally observed expansion of MXene interlayers by 1.1 Å. For Li cations, the difference (≈2.2 Å) is larger than the experimental value (1.19 Å). The difference between size of Cl^−^ anion and diameters of Rb^+^, Cs^+^ and NH_4_^+^ cations (in dehydrated state) is much smaller (0.6 Å, 0.3 Å, 0.3 Å) which is also in agreement with small changes of interlayer distance of MXene in these electrolytes as a function of applied voltage.

It is also interesting to discuss the relations between the sizes of electrolyte ions (shown in Figure 6b in both hydrated and not hydrated state) and width of the MXene interlayers provided by swelling. The most common method to estimate width of MXene interlayers is to consider a difference (≈2.3 Å) between *d*(001) of vacuum dry state and *d*(001) expanded by swelling in water or electrolyte solutions. However, the 2.3 Å value is too small for insertion of alkali metal cations in hydrated state; it is also too small for insertion of Rb^+^, Cs^+^, and NH_4_^+^ cations even in completely dehydrated state. Assuming intercalation of cations in dehydrated state provides reasonable agreement with changes of *d*(001) in alkali metal salt solutions. Dehydrated cations smaller than 2.3 Å spontaneously intercalate MXene interlayers causing it further expansion while larger cations are size excluded.

An alternative method to calculate the effective width of interlayers would be to use the difference between water swollen state and *d*(001) found for Li‐free MXene (3.7 Å). This type of MXene is synthesized by HF etching and typically shows smaller interlayer distance (≈10.2–10.7 Å).^[^ ^3^, ^4^ ^]^ Assuming that Li intercalated into interlayers after synthesis by LiF + HCl etching is not rigidly attached to MXene layers and participates in processes driven by a change of potential in aqueous electrolyte solutions, the width of the interlayers of Li‐MXene in pure water is about 14.2 − 10.5 Å = 3.7 Å. A similar calculation for estimation of overall interlayer spacing was considered in our in situ study of LiF + HCl synthesis of MXene.^[^ ^21^ ^]^

Assuming ≈10.5 Å as a baseline for calculation of effective width of interlayers in aqueous electrolytes, the expansion of the MXene c‐lattice in LiCl and NaCl solution‐swollen state (≈5.5–6 Å) is closer to the diameter of hydrated cations (7.6 Å and 7.2 Å for Li+ and Na+, respectively).^[^ ^59^ ^]^ The ≈6 Å width of MXene interlayers experimentally observed in electrolyte swollen state is only little smaller than the diameter of Cl^−^ anion in hydrated state (6.6 Å for full hydration). The sizes of hydrated Rb^+^, Cs^+^, and NH_4_^+^ cations are nearly the same (≈6.6 Å) as for hydrated K^+^ and smaller than the size of the Li^+^ and Na^+^ (Figure 6b).^[^ ^59^ ^]^

Replacing hydrated chlorine anions with hydrated alkali metal cations would result in rather small change of *d*(001) not compatible with the experimental data (Figure 6). Note that the diameter of hydrated Cl^−^ anion is smaller than the diameter of hydrated Na+ and Li+ cations. Replacing larger hydrated Li+ and Na+ cations with smaller hydrated chlorine anions would be expected to result in a decrease of *d*(001) instead of the experimentally observed c‐lattice expansion. Therefore, replacement of hydrated anions with hydrated cations is not in agreement with the changes of *d*(001) observed experimentally even if we assume larger width of MXene interlayers (3.7 Å in pure water).

The question remains which method of MXene interlayer width calculation is better: considering as a baseline the “dry” water‐free state (≈14 Å) or broader width with baseline at Li‐free state (≈12.5 Å). As discussed above, the width of interlayers calculated as a difference between “dry” state and water swollen state (2.3 Å) is too small to enable entrance of cations in hydrated state and for anions to enable entrance even in dehydrated state. The size of MXene interlayers calculated as a difference between Li‐free state (≈12.5 Å) and water swollen state is closer to the diameter of hydrated ions. However, intercalation of hydrated ions does not explain experimentally observed changes in MXene inter‐layer width as a function of applied potential but is in relatively good agreement with overall expansion of MXene lattice in electrolyte solutions.

It should be noted that the difference between *d*(001) observed at +1 and −1 V was considered above so far only for both hydrated and not hydrated cations and anions. However, assuming that intercalated anions are completely hydrated and cations are partly dehydrated would explain the observed expansion of MXene structure under positive polarization.

We note that several earlier studies reported that anions are not capable to intercalate MXene interlayers in principle assigning it to electrostatic repulsion from negatively charged 2D sheets.^[^ ^39^ ^]^ Assuming the absence of anions intercalation into MXene interlayers, the difference between the *d*(001) value recorded at +1 and −1 V (Figure 6b) could be interpreted as a partial withdrawal and reinsertion of only cations. Complete withdrawal of Li^+^, Na^+^, or K^+^ cations from MXene interlayers is expected to result in a *d*(001) value of ≈14 Å typical for swelling of MXene in pure water. Significantly smaller changes of *d*(001) found experimentally could possibly be assigned then to incomplete withdrawal of cations. However, this interpretation is not compatible with the charge balance condition in a supercapacitor with symmetrical electrodes. Absence of anions intercalating one electrode is expected to result in the absence of cations intercalation on another electrode in symmetric setup, thus making operation of supercapacitors impossible. Both our data and many earlier studies demonstrate successful operation of MXene supercapacitors with a variety of aqueous electrolytes. Moreover, intercalation of anions in the process of supercapacitor operation was explicitly reported in several studies.^[^ ^38^ ^]^ We note that it is difficult to compare our data recorded from a parallel plate supercapacitor, with data recorded in three‐electrode cells with a carbon material as the counter ‐electrode.^[^ ^39^ ^]^ A small change of interlayer distance was observed in experiments with extremely concentrated 14M LiCl but in the opposite direction (14.7 Å at −0.3 V to 14.3 Å at 0.8 V) and was assigned to extraction of intercalated Li cations with the absence of anions intercalation. The water‐in‐salt LiCl electrolyte is likely to be more similar to ionic liquids in terms of electrolyte migration as a function of applied potential. The 1M LiCl electrolyte used in our experiments can be considered as two‐component system with ions carrying the charge and water as a medium remaining between MXene layers even after withdrawal of all electrolyte ions. Therefore, we believe that the absence of anion intercalation is likely not general for all MXene supercapacitors.

In our opinion, the replacement of anions with cations is still the most likely explanation of MXene's *d*(001) changes observed in our experiments with 1M LiCl, 1M NaCl, and 1M KCl electrolytes as a function of applied potential (Figure 3, 4, and 6). However, the data also indicate that negatively charged MXene sheets do provide a hindrance for intercalation, as it is especially clear in the XRD “heat map” for the NaCl electrolyte. Complete conversion of MXene structure into the expanded anion intercalated phase is found only at higher values of applied voltage (close to +1 V), while the coexistence of two (001)‐reflections is found at lower voltage values.

Much stronger swelling of MXene in LiCl, NaCl, and KCl electrolytes (both with and without applied voltage) compared to swelling in RbCl, CsCl, and NH_4_Cl could be anticipated to result in significant difference of supercapacitor performance. The expansion of *d*(001) as compared to swelling of MXene in pure water is commonly considered as evidence of cation intercalation^[^ ^3^, ^63^ ^]^ while the absence of expansion indicates also the absence of cation intercalation. Surprisingly, MXene supercapacitors with all chloride salt electrolytes showed similar performance. The CV loops recorded prior to the operando tests in 0–0.6 V voltage window and directly during operando cycles in 0.0–1.0 V show only little smaller energy storage performance for RbCl, CsCl, and NH_4_Cl electrolytes (Figure 2). Unexpected absence of correlation between the width of MXene interlayers (and changes of this width as a function of applied voltage) recorded by XRD in chloride salt electrolytes was confirmed in a set of experiments performed with standard (Swagelok) supercapacitor cells. Detailed electrochemical characterization of MXene supercapacitors with H_2_SO_4_, LiCl, NaCl, KCl, RbCl, CsCl and NH_4_Cl electrolytes was performed in 0 to 0.6 V voltage window (Figure S15–S22, Supporting Information). The specific capacitance values recorded at slow charge–discharge (comparable to operando experiments) demonstrated relatively weak decreasing trend for the set of alkali metal electrolytes (62.8 F g^−1^, 50.1 F g^−1^, 56.8 F g^−1^, 49.9 F g^−1^, and 44.9 F g^−1^ for 1M LiCl, NaCl, KCl, RbCl, and CsCl, respectively, at 0.03 A g^−1^ scan rate). There is no sharp drop in energy storage parameters of MXene supercapacitors in RbCl, CsCl, and NH_4_Cl electrolytes as it could be anticipated considering *d*(001) values similar to pure water and the width of interlayers (provided by swelling) seemingly not sufficient for accommodation of cations and anions.

Therefore, our experiments reveal paradoxical absence of obvious correlations between changes of MXene structure in electrolyte solutions and energy storage in supercapacitors.

Summarizing this discussion, the changes of MXene structure observed inside supercapacitors need to be considered as a complex effect related to the expansion and contraction of interlayer spacing at both electrodes. To the best of our knowledge, this study is the first to analyze changes of MXene structure inside fully assembled supercapacitors and at both electrodes. The changes of the MXene interlayer spacing in alkali metal chloride electrolytes are not always correlated with the size of cations and anions (in either hydrated or not hydrated state) or the difference between the size of cations and anions (when potential is switched between positive and negative). At least partial dehydration of cations and anions is likely required to explain experimental observations. Comparing the size of ions with the width of MXene interlayers expanded by swelling in electrolyte solutions raises new questions about the mechanism of supercapacitor energy storage.

Possibly the most surprising finding of this study is that supercapacitors can be successfully charged and discharged with the interlayer spacing of MXene electrodes being clearly smaller than the size of cations and anions in hydrated state (e.g., in RbCl, CsCl, and NH_4_Cl electrolytes). Moreover, energy storage parameters of MXene supercapacitors do not show obvious correlations with changes of *d*(001).

In this respect, one needs to consider the possibility that a *major* part of energy storage in MXene supercapacitors is not related to the intercalation of ions into MXene interlayers. Hypothetically, the energy storage in MXene electrodes can be related to few‐layered flakes with major contribution to charge storage related to their external surface rather than to the penetration of ions into interlayers and the internal surface area, similar to supercapacitors based on rGO. In the absence of swelling in electrolyte solutions, the energy storage in rGO supercapacitors is related to relatively large outer surface area or few‐layered flakes (100–700 m^2^ g^−1^).^[^ ^64^, ^65^ ^]^ XRD recorded from rGO electrodes shows c‐lattice typical for graphitic carbons with interlayer distance close to 3.4 Å, too small for penetration of ions. Remarkably, the study of MXene electrode hydration, which also included electrodes intercalated with some alkali metal cations, reported the size of interspaces filled with water significantly higher (20–30 Å) than the size of MXene interlayers in the stacked structure (3–7 Å).^[^ ^42^ ^]^ It is important to note that MXene was prepared in this study using the same method as in our experiments (LiF + HCl etching) and possibly had similar swelling properties (for the crystalline part) in alkali metal chloride electrolytes.^[^ ^42^ ^]^ Migration of anions and cations within larger spaces between few‐layered MXene flakes (similar to rGO) would explain experimentally observed data, such as similar performance of MXene electrodes in all alkali metal electrolytes, including for example, RbCl, CsCl, and NH4Cl where no obvious intercalation of both cations and anions could be detected by XRD.

However, the MXene which we used in our experiments showed rather small BET surface area below 10 m^2^ g^−1^, much smaller than in rGO supercapacitors (≈100–500 m^2^ g^−1^). Small BET surface area suggests that a significant contribution to energy storage from intercrystalline spaces is unlikely, but still cannot be completely ruled out. Immersing electrodes into solution might change even the intercrystallite spaces width as compared to solvent‐free state and larger external surface area of few‐layered MXene flakes cannot be completely ruled out. If this idea is true, preventing 2D sheets of MXene from restacking in the process of electrode deposition from dispersions should be of main importance for increasing energy storage capacity rather than engineering access of MXene interlayers for easier penetration of ions.

## 5 Conclusions

In situ and operando synchrotron radiation XRD experiments were performed with Ti‐MXene symmetrical parallel plate supercapacitors assembled inside of specially designed micro capillary cell. The structure of both MXene electrodes was studied as a function of voltage applied to working electrode (0 to 1 V and 0 to −1 V) in alkali metal chloride electrolytes (LiCl, NaCl, KCL, RbCl, and CsCl) and NH_4_Cl electrolyte in order to verify the effects of cation size. Additional tests were also performed with H_2_SO_4_, as it is the electrolyte most used in MXene supercapacitors. The main results of these experiments are summarized as follows: 1) The c‐lattice parameter of MXene evaluated using *d*(001) expands by maximal ≈3–3.5 Å (relative to saturated water swollen state) in LiCl, NaCl, and KCl electrolytes when a voltage up to 1 V is applied. The *d*(001) value observed for MXene in RbCl, CsCl, and NH_4_Cl remains close to the value found in pure water. 2) Strong and reversible changes of MXene interlayer distance as a function of applied voltage (+1 and −1 V) were found in LiCl, NaCl, and KCl electrolytes (≈1.1–1.8 Å); 3) The MXene lattice expands when positive potential is applied to electrode and contracts when the voltage is decreased back to 0 V. This effect is assigned to insertion of larger Cl^−^ anions replacing smaller cations in the MXene interlayers. The difference in *d*(001) value observed at 1 and −1 V is likely to correspond to the difference between the size of inserted anions and cations. 4) The structure of MXene in NaCl electrolyte shows sharp transitions between two distinctly different states with a *d*(001) value difference of ≈1.8 Å at 0 and 1 V (at the positive electrode) corresponding well to the difference between diameters of dehydrated Cl^−^ anions and Na^+^ cations. The changes of *d*(001) as a function of applied voltage are more gradual for MXene in LiCl and KCl electrolytes indicating effects of random interstratification in intercalation of interlayers. 5) No expansion of MXene interlayers was found in CsCl, NH_4_Cl (and minor changes found in RbCl) electrolytes. The interlayer spacing of MXene in these electrolytes is inconsistent with the insertion of hydrated ions. 6) Stable MXene supercapacitor performance with rather small *d*(001) variations was observed in H_2_SO_4_ electrolyte over tens of cycles controlled by operando XRD. The difference in *d*(001) observed as a function of applied voltage (≈0.7 Å) suggest that the size of sulphate anions is larger than the size of cations (H_3_O^+^). 7) Analysis of experimental data shows that calculation of the MXene interlayer width as a difference between water‐swollen and vacuum‐dried states provides too small value insufficient for penetration of anions even in completely dehydrated state. Calculation based on the difference between water‐swollen structure and Li‐free MXene structure provides the width of the MXene interlayers sufficient for accommodation of alkali metal cations and Cl^−^ anions in hydrated state.

Our experiments revealed effects related to the match or mismatch between the size of interlayers and size of electrolyte cations/anions inside operating supercapacitors in the process of charging and discharging. Electrostatic repulsion of anions and negatively charged 2D sheets is likely to contribute to stronger expansion of MXene interlayers at the anion intercalated electrodes, while for cation intercalated electrodes the lattice expansion is similar in LiCl, NaCl, and KCl electrolytes.

Possibly the most important result of our study is paradoxical absence of obvious correlations between changes of MXene structure in electrolyte solutions (with or without applied potential) and energy storage in supercapacitors with chlorides as electrolytes. One of the most puzzling effects observed in our experiments is the successful operation of MXene‐based supercapacitors with small width of swelling‐expanded interlayers not allowing penetration of both cations and anions in hydrated state (e.g., in CsCl and NH_4_Cl electrolytes).

Partial or complete dehydration of ions is likely needed in order to explain all experimental results. Alternatively, major part of energy storage in MXene supercapacitors could be related not to intercalation of interlayers in crystalline stacked structure but (similar to rGO) to external surface of few‐layered MXene flakes.

Finally, it should be noted that the results presented in our study might also be useful in applications other than supercapacitors. For example, lattice expansion of MXene in solutions of some salts and the absence of this expansion in other salts might be useful for applications related to selective sorption and sorption‐related separation of complex solution mixtures. Selective size‐related penetration of ions into MXene interlayers could also be promising for sensors or membrane separation applications.

## Supporting Information

Supporting Information is available from the Wiley Online Library or from the author.

## Conflict of Interest

The authors declare no conflict of interest.

## Supporting information

Supplementary Material

## Data Availability

The data that support the findings of this study are available from the corresponding author upon reasonable request.
