# Supplementary material for: Operando X‐Ray Diffraction Study of MXene Electrode Structure in Supercapacitors with Alkali Metal Electrolytes
Source: Small Sci. 2025 Oct 14;5(12):e202500367. doi: 10.1002/smsc.202500367 (PMC12697801; doi:10.1002/smsc.202500367)
Supplement: Supplementary file 1 — Supplementary Material [file SMSC-5-e202500367-s001.pdf]

Supporting information.

Operando XRD study of MXene electrodes structure in supercapacitors with alkali metal electrolytes.

Guil Li,[1] Nicolas Boulanger,[1] Bartosz Gurzeda, Susu Bi, Christoph Hennig[2,3], Alexandr V. Talyzin[1]*

[1] Department of Physics, Umeå University, Umeå S-90187, Sweden.

*e-mail: alexandr.talyzin@umu.se

[2] Rossendorf Beamline (BOBL-BM20) a tEuropean Synchrotron Radiation Facility (ESRF), ,

71 Avenue des Martyrs, 38000 Grenoble , France

[3] Helmholtz-Zentrum Dresden-Rossendorf, Insititute of Resource Ecology, Bautzner Landstrasse 400, 01328 Dresden, Germany

This file includes following data:

1. Characterization of MXene sample used in synchrotron radiation XRD experiments described in the main part. Figures S1-S6
2. XRD data recorded in situ at static conditions following step like increase and decrease of voltage applied to working electrode. Figures S7-S12
3. XRD data recorded from MXene electrode in RbCl electrolyte as a function of potential cycled between +1.0V and -1.0 V and CV curves recorded during cycling. Figure S13
4. XRD data and CV loops recorded from MXene electrode in NH4Cl electrolyte over prolonged cycling, 19 cycles between 0.0V and +1.0 V. Figure S14
5. Characterization of standard supercapacitors with MXene electrodes in alkali metal chloride salts and NH_4Cl. Figures S15-S22.

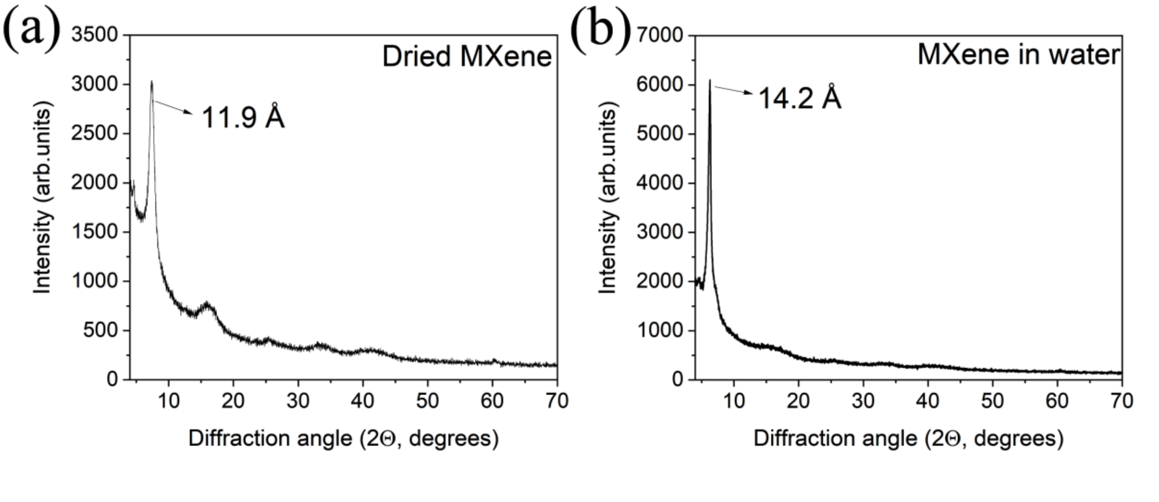

Figure S1. XRD patterns of dried MXene (a) and MXene in water (b).

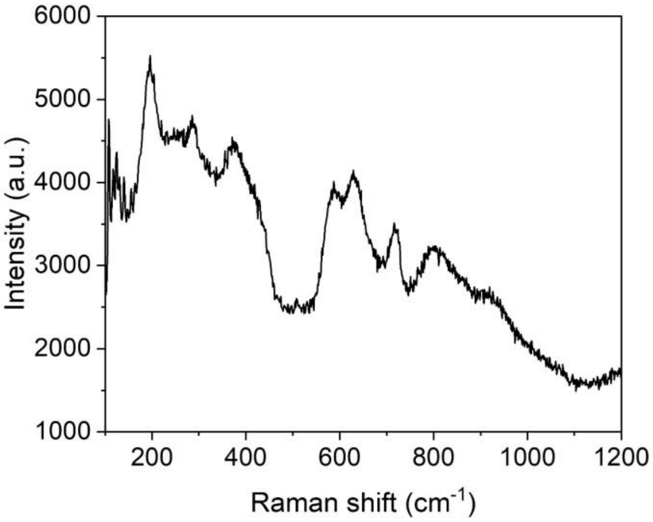

Figure S2. Raman spectrum of dry MXene.

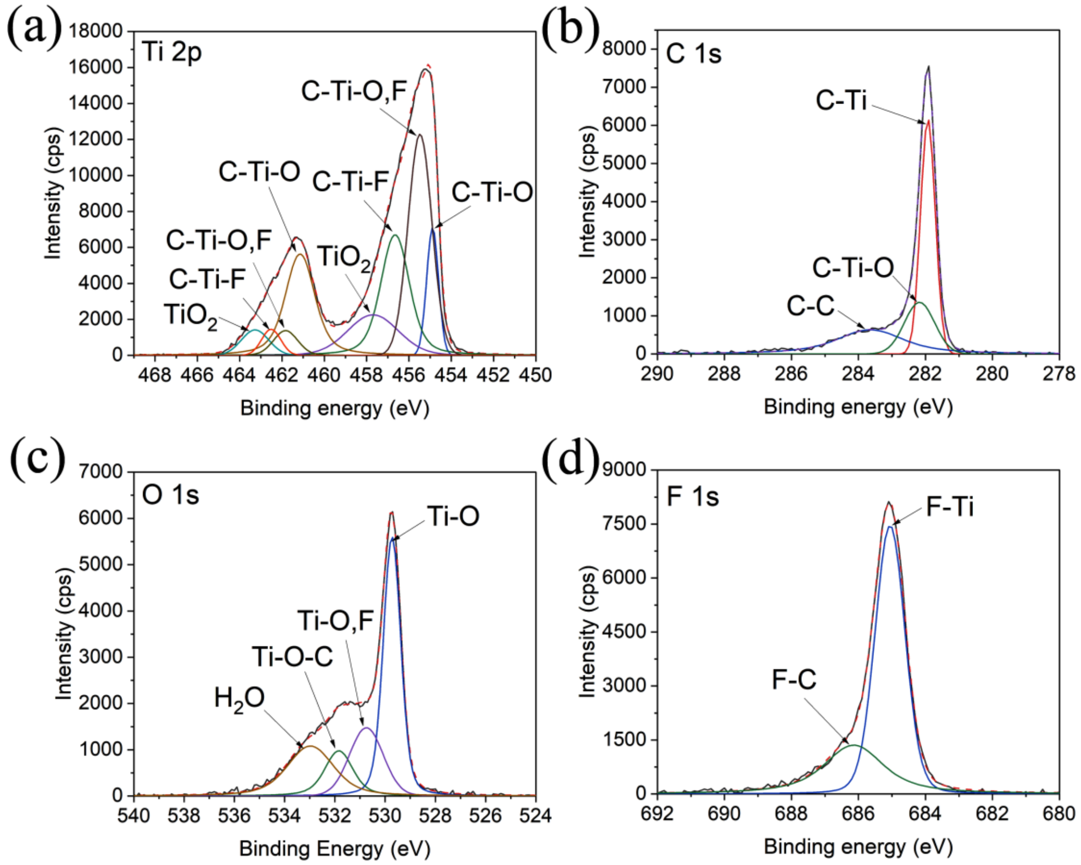

Figure S3. XPS results of dry MXene, Ti2p (a), C1s (b), O1s (c), F1s (d).

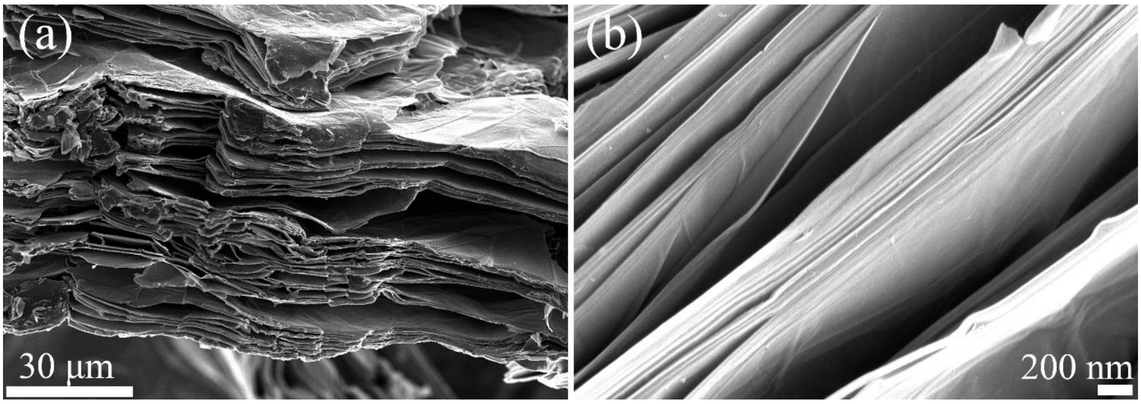

Figure S4. SEM pictures of dry MXene.

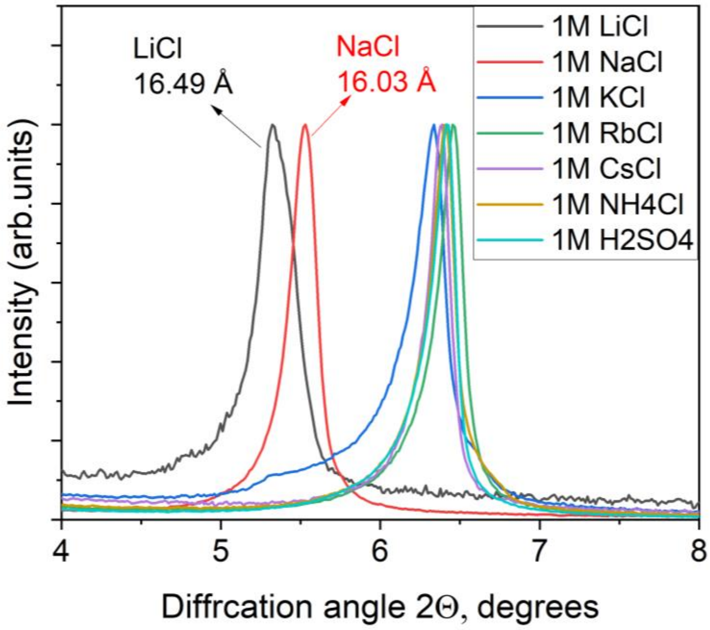

Figure S5. XRD patterns (CuKα radiation) recorded from MXene immersed in electrolyte solutions.

Experiments with electrolyte immersed MXene were performed using different batch prepared using identical procedure. The d(001) values recorded from this MXene in dry state and in water immersed state were 11.5Å and 13.7Å respectively (~2.2 Å difference). The d(001) of electrolyte immersed MXene remained similar to water immersed value for KCl (13.97Å), RbCl (13.69 Å), CsCl (13.84Å), NH4Cl (13.99 Å), H2SO4 (13.78Å).

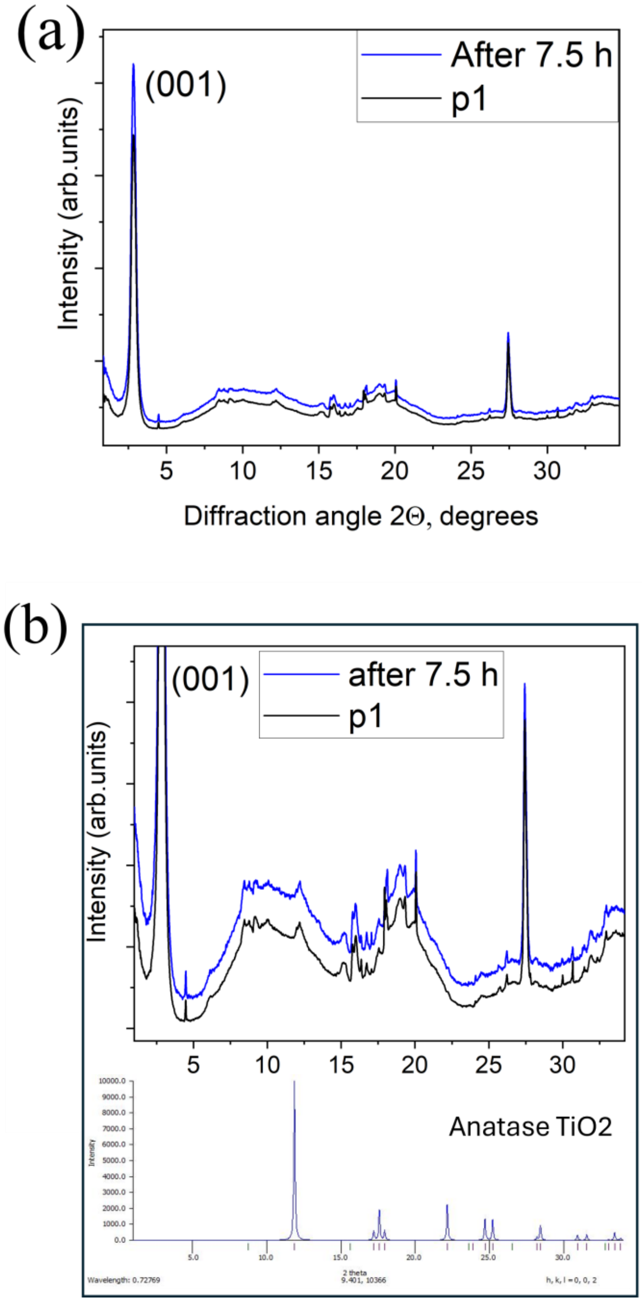

Figure S6. XRD patterns recorded from MXene electrode at the starting time point and after prolonged charge-discharge cycling at working electrode in 0V to 1V voltage window (1M H_2SO_4). (see Fig.2 d). a) with full scale, b) zoomed to show low intensity reflections compared to simulated theoretical XRD pattern of anatase TiO_2. The broad features are from plastic capillary cell.

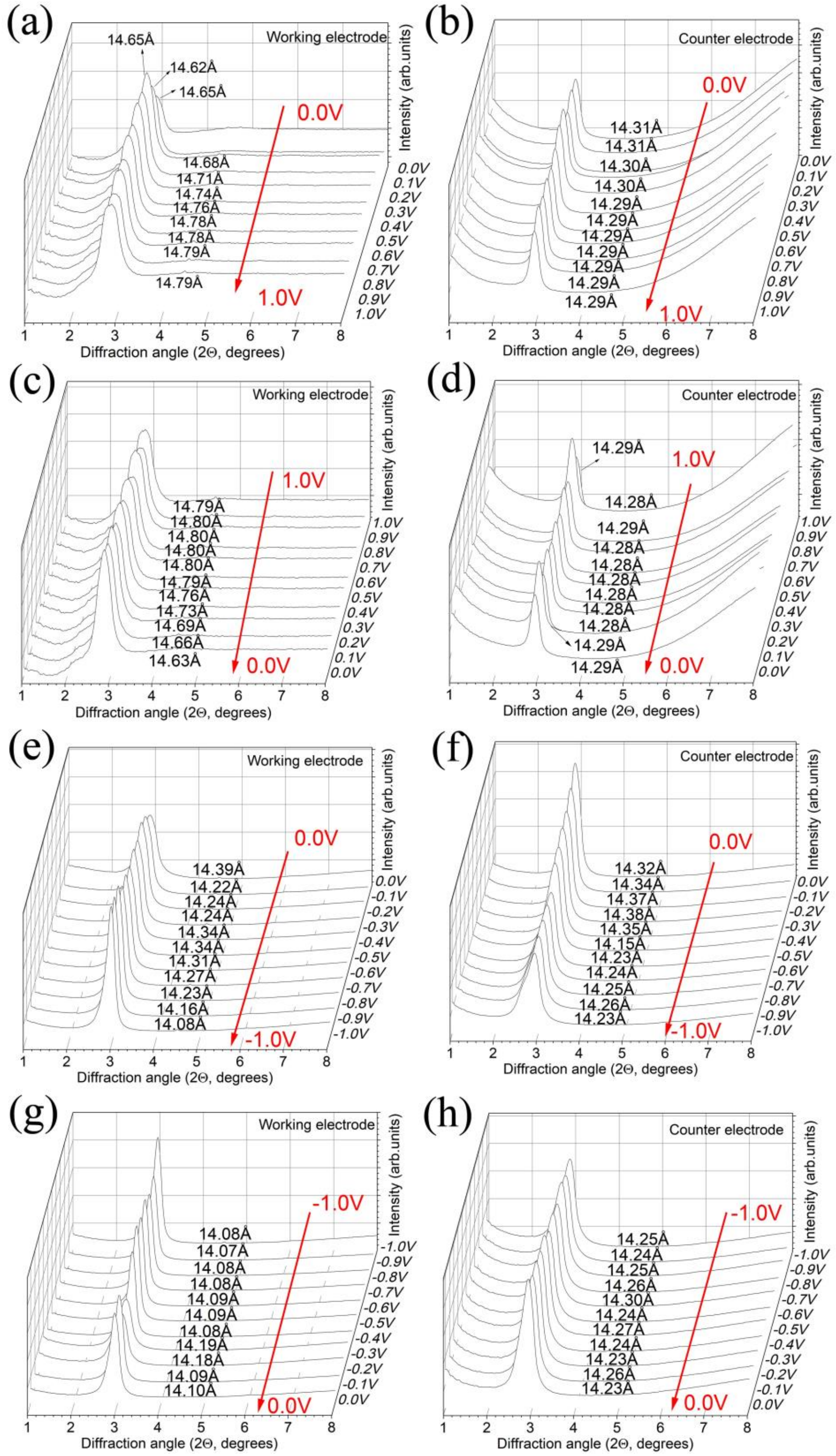

Figure S7. XRD patterns of MXene working electrode (a, c, e, g) and counter (b, d, f, h) in 1M H2SO4 electrolyte with applied voltage going from 0 V to 1 V and -1 then back to 0V.

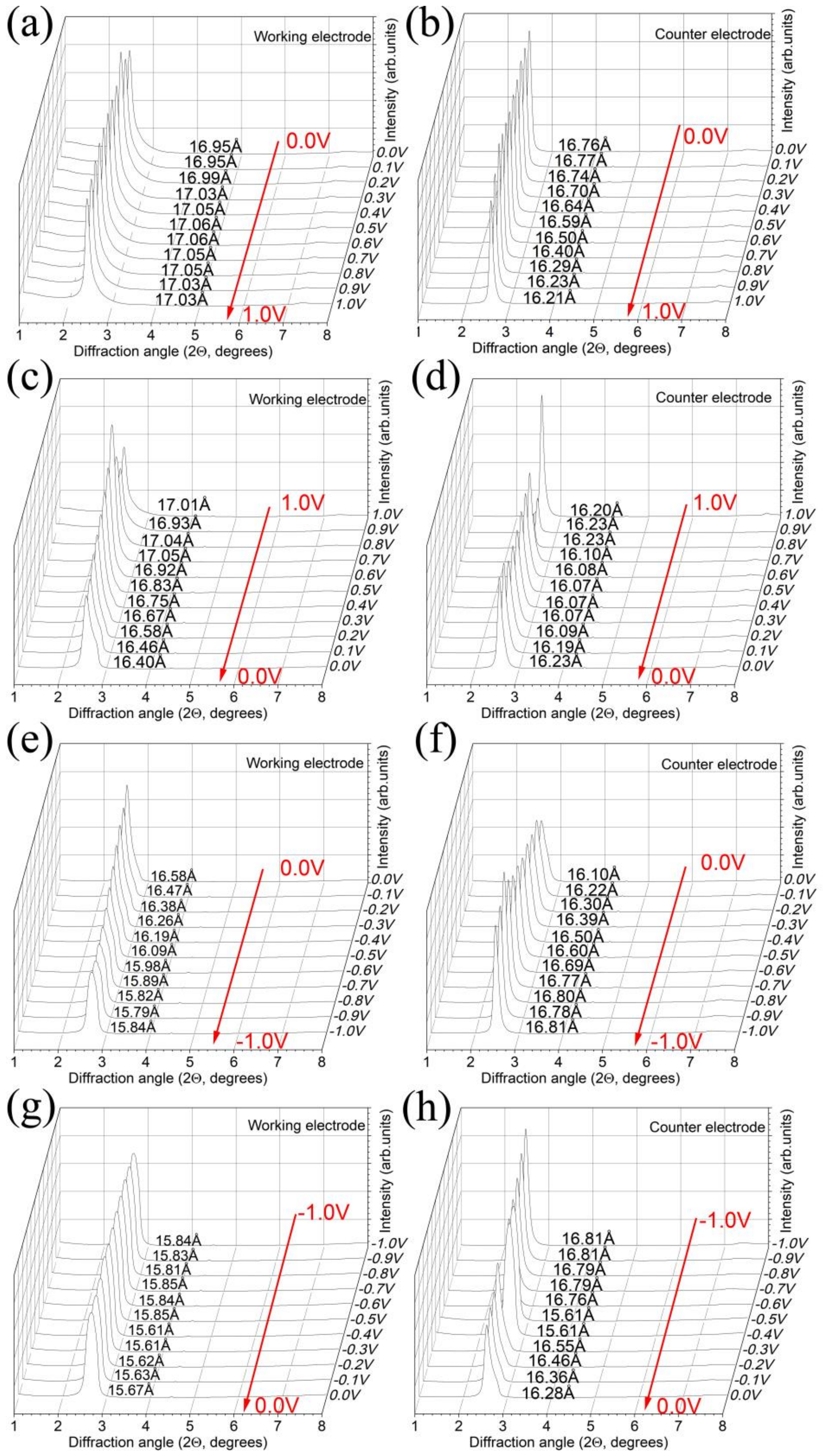

Figure S8. XRD patterns of MXene working electrode (a, c, e, g) and counter (b, d, f, h) in 1M LiCl electrolyte with applied voltage going from 0 V to 1 V and -1 then back to 0V.

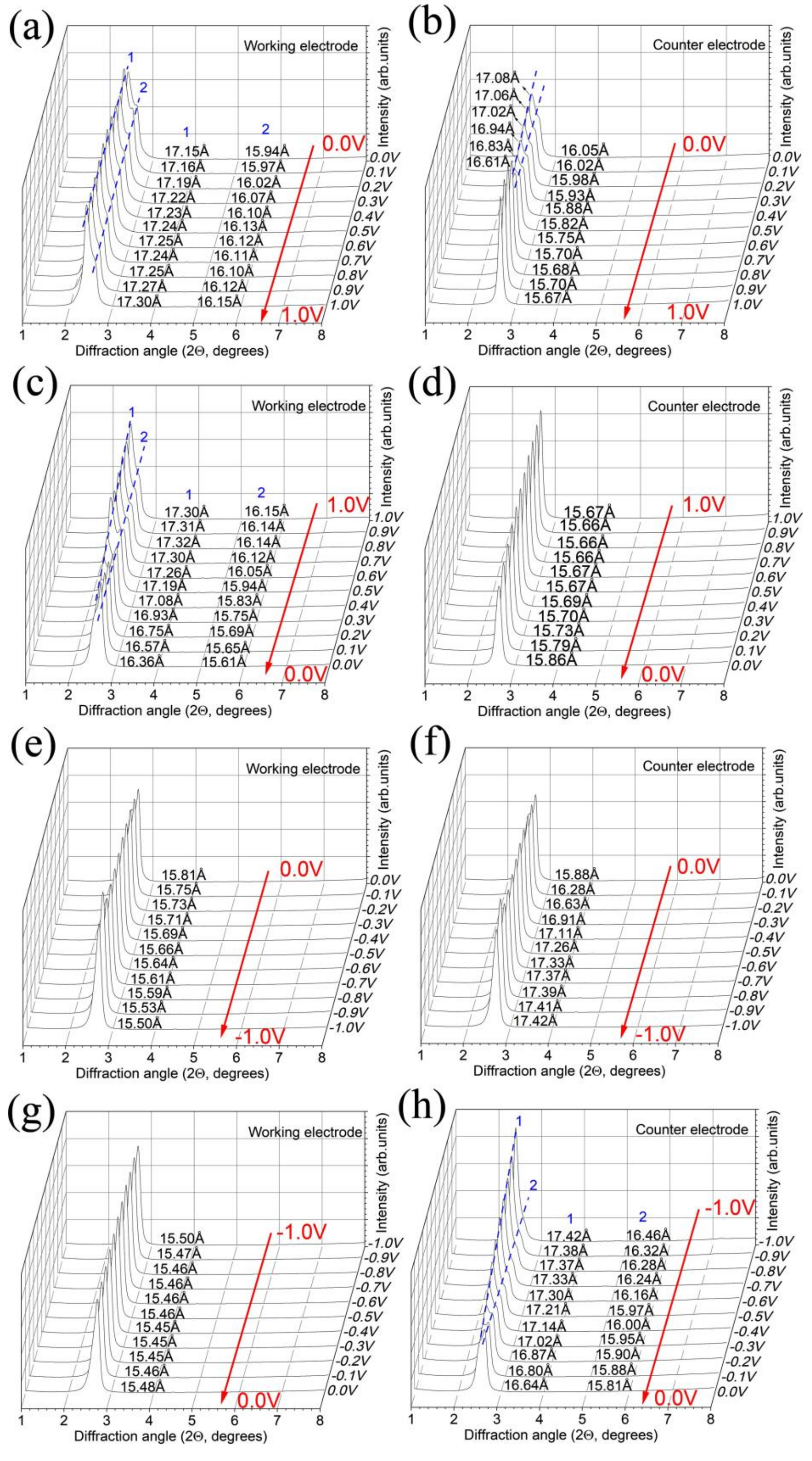

Figure S9. XRD patterns of MXene working electrode (a, c, e, g) and counter (b, d, f, h) in 1M NaCl electrolyte with applied voltage going from 0 V to 1 V and -1 then back to 0V.

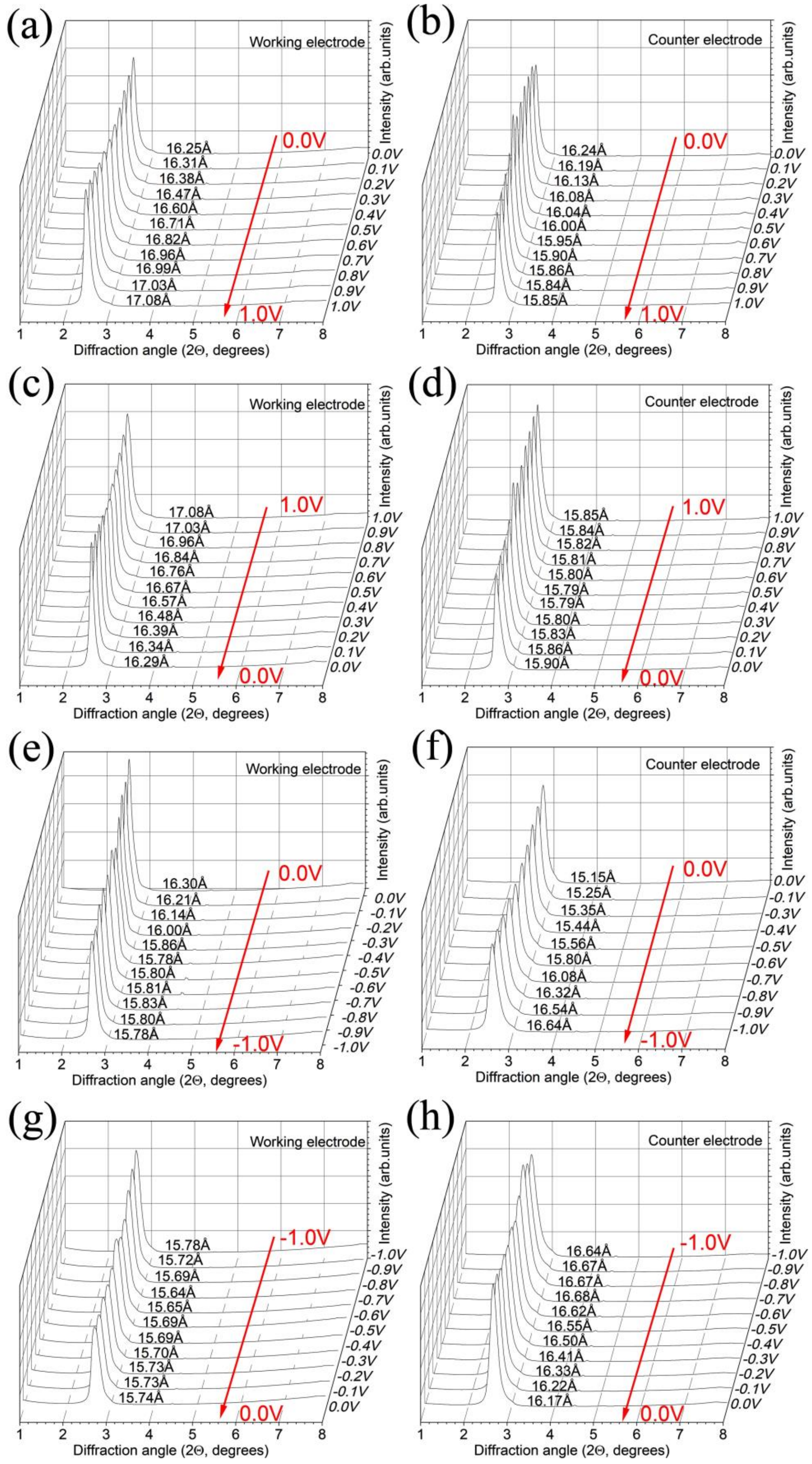

Figure S10. XRD patterns of MXene working electrode (a, c, e, g) and counter (b, d, f, h) in 1M KCl electrolyte with applied voltage going from 0 V to 1 V and -1 then back to 0V.

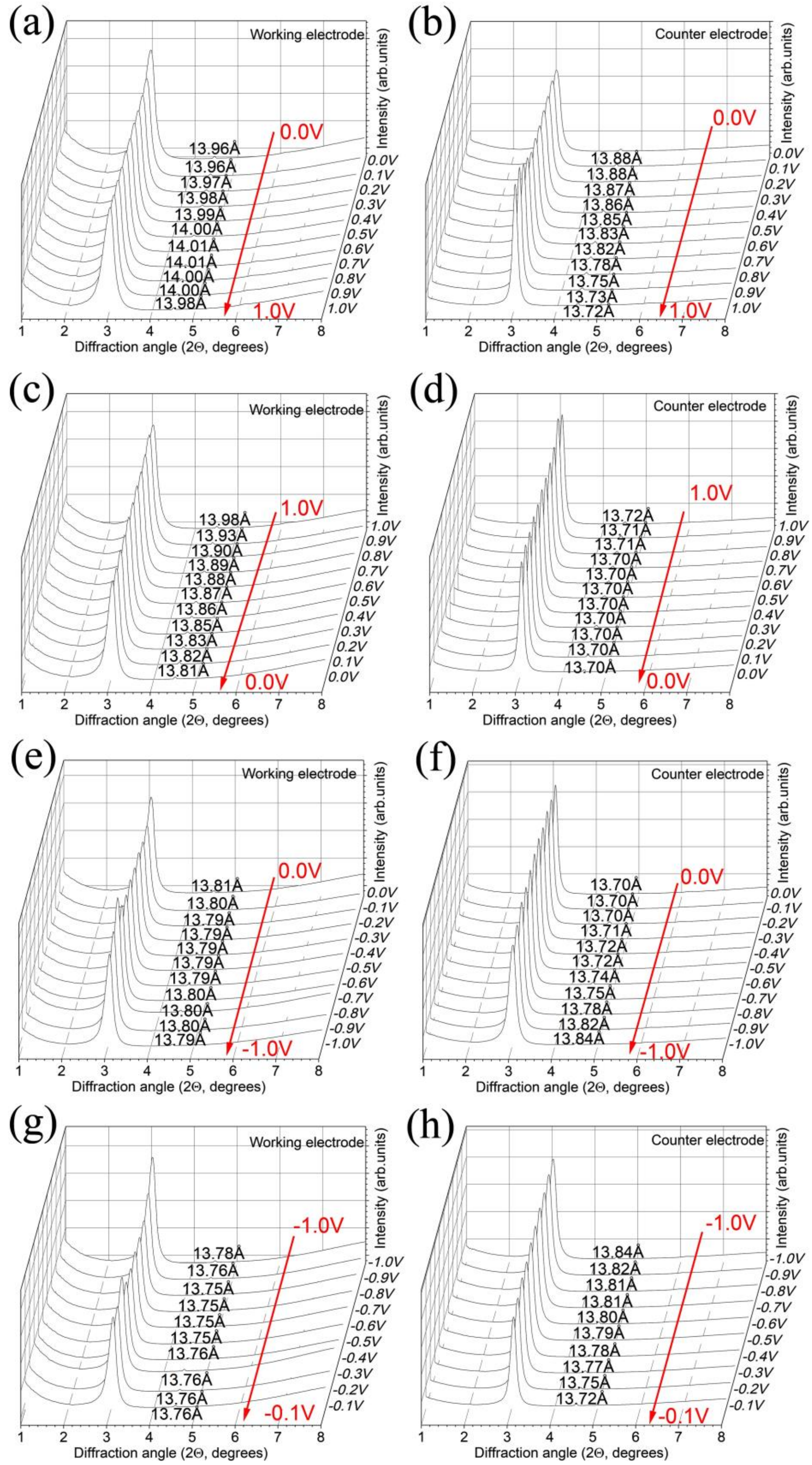

Figure S11. XRD patterns of MXene working electrode (a, c, e, g) and counter (b, d, f, h) in 1M CsCl electrolyte with applied voltage going from 0 V to 1 V and -1 then back to 0V.

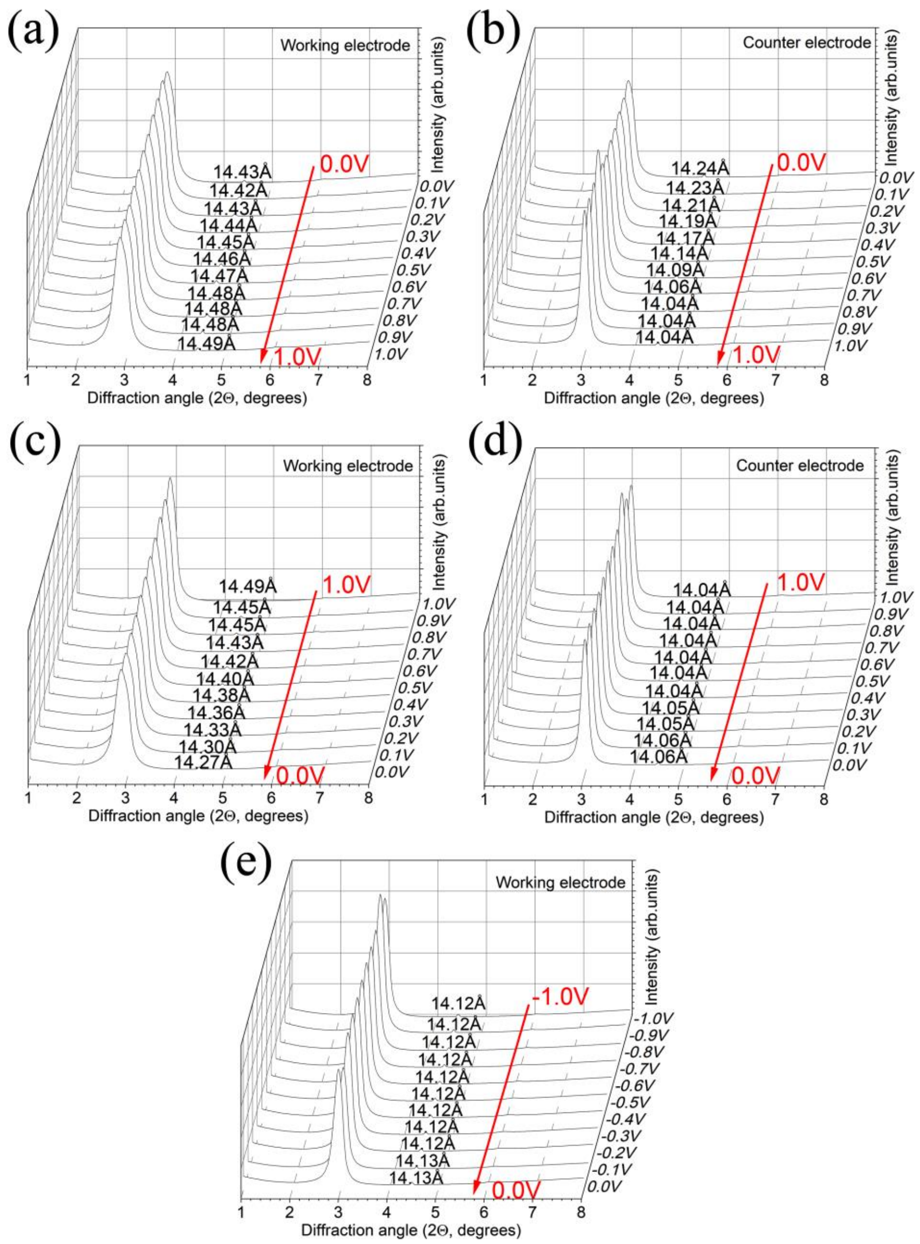

Figure S12. XRD patterns of MXene working electrode (a, c) and counter (b, d,) in 1M NH4Cl electrolyte with applied voltage going from 0 V to 1 V and back to 0V. e) data recorded from working electrode with change of applied voltage from -1V to 0V.

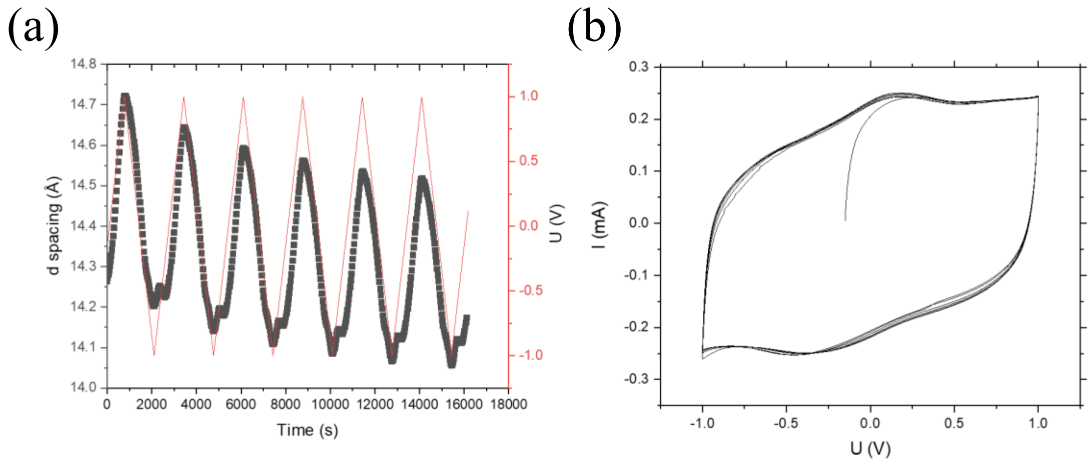

Figure S13. (a) Interlayer spacing d(001) of MXene electrode in RbCl electrolyte as a function of potential cycled between +1.0V and -1.0 V; (b) CV curves recorded during cycling.

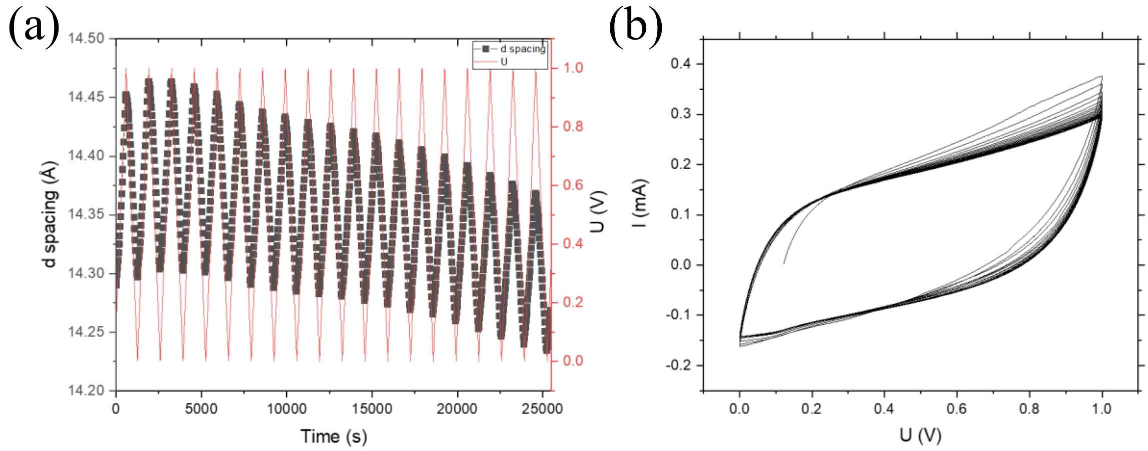

Figure S14. (a) Interlayer spacing d(001) of MXene electrode in NH_4Cl electrolyte as a function of potential cycled between 0.0V and +1.0 V; (b) CV curves recorded during cycling.

Experiments with standard supercapacitors.

Preparation and characterization of devices.

60mg MXene powder was added in to 3 ml DI water and followed by 3 hours sonication. Then 0.3 ml of the MXene dispersion taken by automatic pipette (1ml) was deposited on the surface of current collectors with an area of 1.2 cm^2 directly and dried in the air. The weight of each electrode was about 6mg. After drying, each two current collectors and a glass fiber membrane separator (Whatsman) were used to assemble a symmetric supercapacitor cell. Cyclic voltammetry (CV), Galvanostatic charge and discharge cycling (CD), electrochemical impedance spectroscopy (EIS) were performed on Iviumstat potentiostat for 1M LiCl, NaCl, KCl, RbCl, CsCl, NH_4Cl, H_2SO_4). For EIS, 10 mV sinusoidal amplitude alternating voltage at frequencies from 0.1Hz to 100000Hz was applied.

MXene sample used for experiments with H_2SO_4 electrolyte was from the same batch as the one used in the in-situ XRD measurements with test performed briefly after the main experiments. For other electrolytes, new batch of MXene was synthesized using identical procedure in order to make characterization using freshly prepared material and to avoid effects of ageing related degradation).

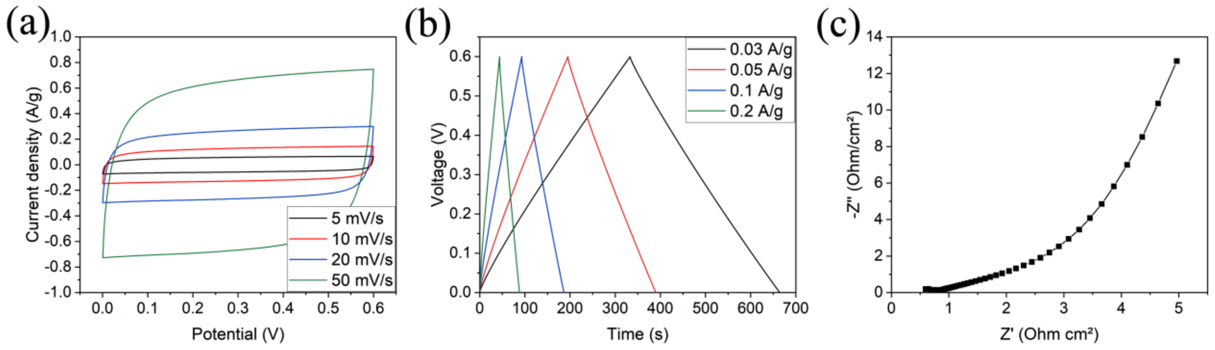

Figure S15. (a) CV, (b)CD and (c) Nyquist plot obtained from EIS for MXene electrodes in 1M LiCl elctrolyte.

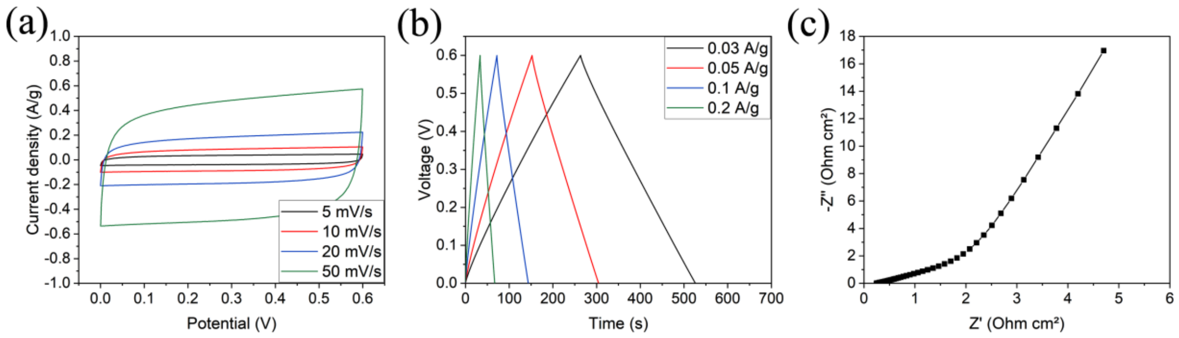

Figure S16. (a) CV, (b) charge-discharge (CD) plot and (c) Nyquist plot obtained from EIS for MXene electrodes in 1M NaCl electrolyte.

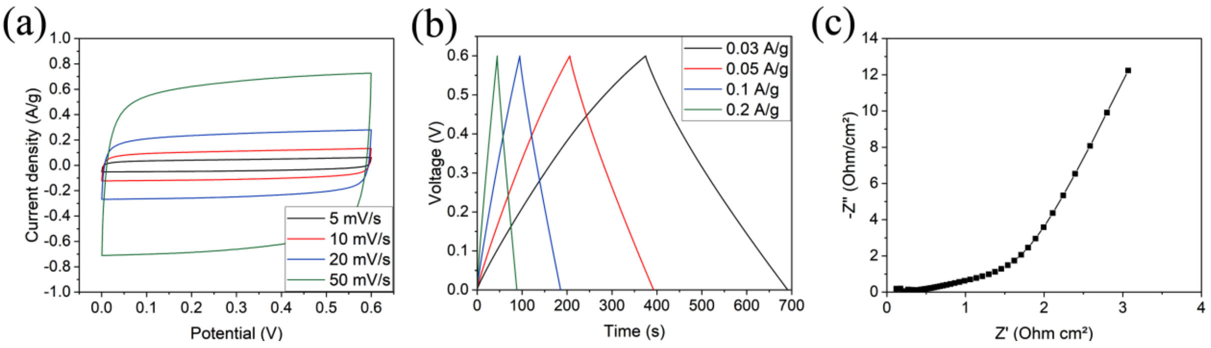

Figure S17. (a) CV, (b)CD and (c) Nyquist plot obtained from EIS for MXene electrodes in 1M KCl elctrolyte.

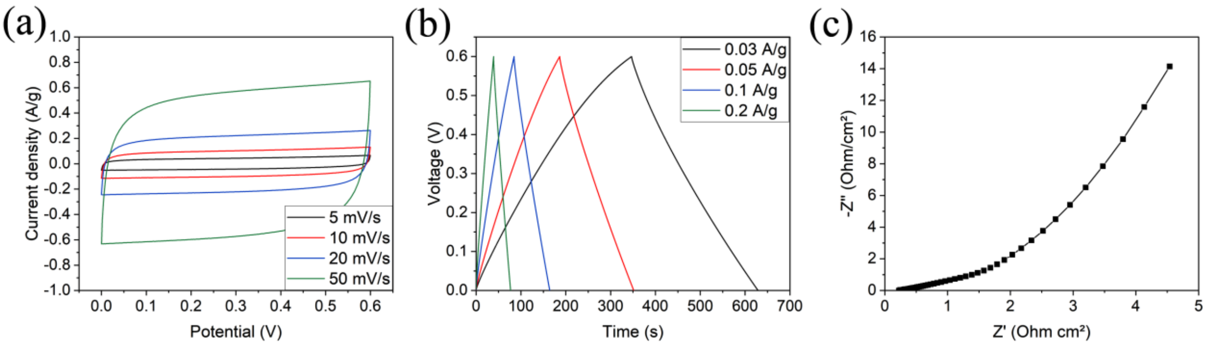

Figure S18. (a) CV, (b)CD and (c) Nyquist plot obtained from EIS for MXene electrodes in 1M RbCl elctrolyte.

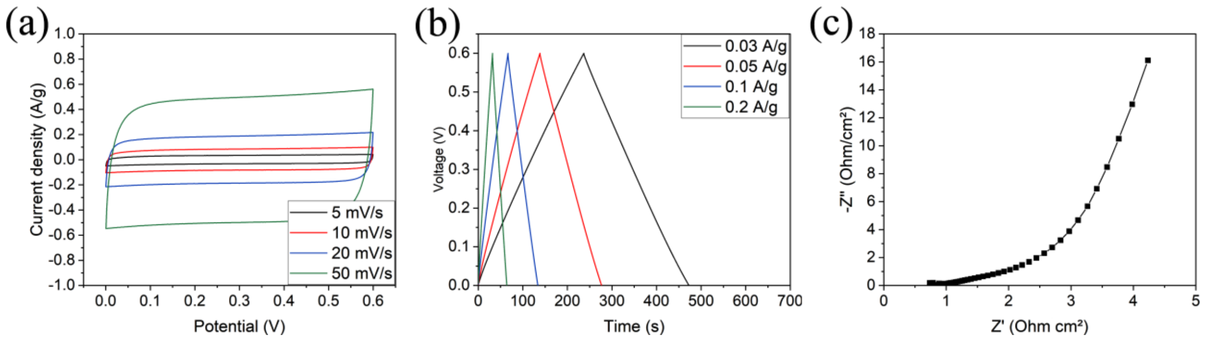

Figure S19. (a) CV, (b)CD and (c) Nyquist plot obtained from EIS for MXene electrodes in 1M CsCl elctrolyte.

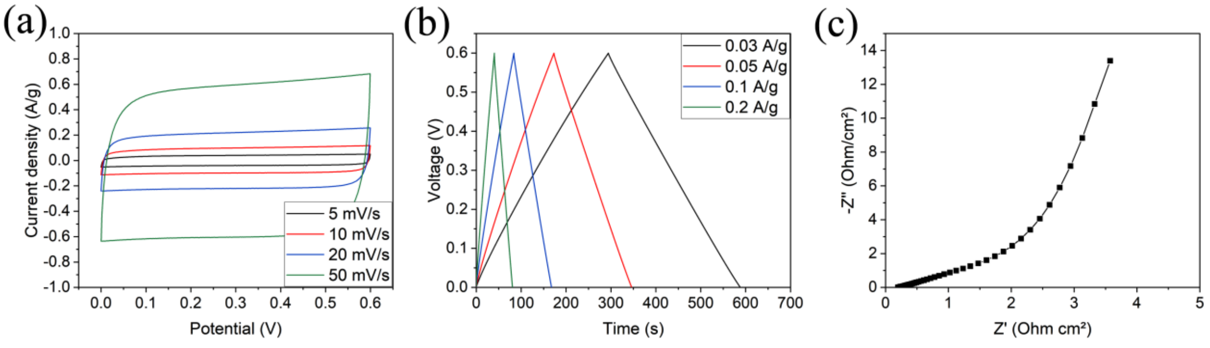

Figure S20. (a) CV, (b)CD and (c) Nyquist plot obtained from EIS for MXene electrodes in 1M NH_4Cl elctrolyte.

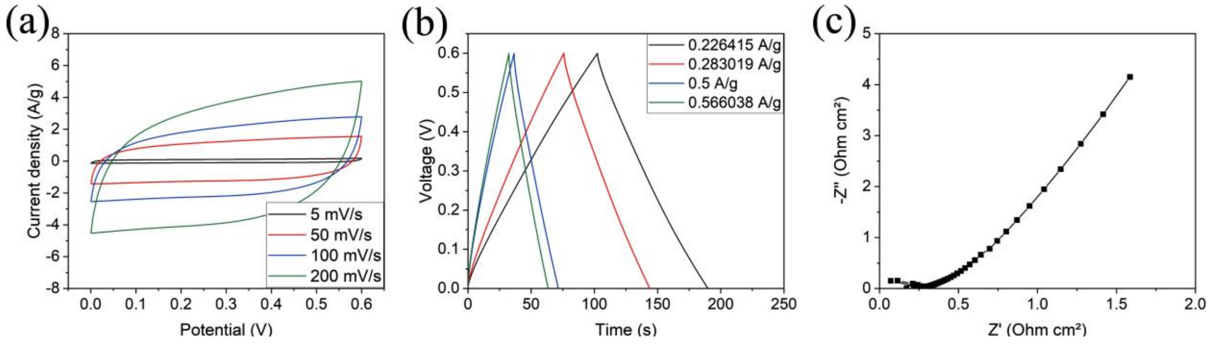

Figure S21. (a) CV, (b)CD and (c) Nyquist plot obtained from EIS for MXene electrodes in 1M H_2SO_4 elctrolyte.

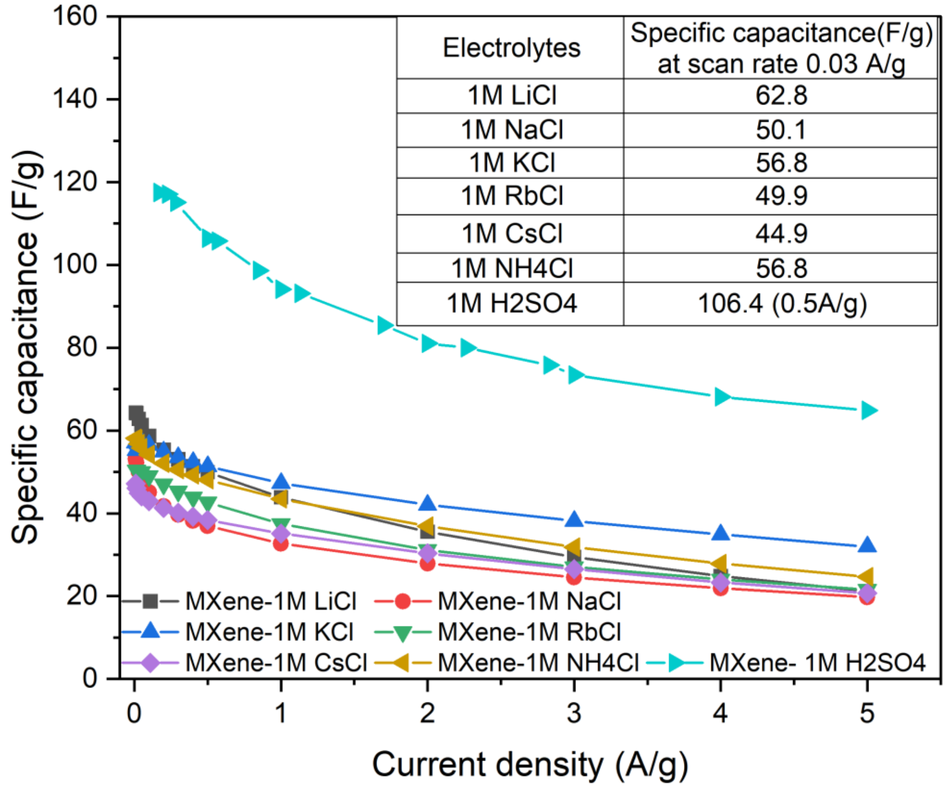

Figure S22. Specific capacitance vs current density for MXene in 1M alkali metal chloride salts and NH_4Cl.
